# Oxidative stress and protein damage responses mediate artemisinin resistance in malaria parasites

**DOI:** 10.1371/journal.ppat.1006930

**Published:** 2018-03-14

**Authors:** Frances Rocamora, Lei Zhu, Kek Yee Liong, Arjen Dondorp, Olivo Miotto, Sachel Mok, Zbynek Bozdech

**Affiliations:** 1 School of Biological Sciences, Nanyang Technological University, Singapore; 2 Mahidol-Oxford Tropical Medicine Research Unit, Faculty of Tropical Medicine, Mahidol University, Bangkok, Thailand; 3 Medical Research Council (MRC) Centre for Genomics and Global Health, University of Oxford, Oxford, United Kingdom; 4 Columbia University Medical Center, New York, New York, United States of America; Dominican University of California, UNITED STATES

## Abstract

Due to their remarkable parasitocidal activity, artemisinins represent the key components of first-line therapies against *Plasmodium falciparum* malaria. However, the decline in efficacy of artemisinin-based drugs jeopardizes global efforts to control and ultimately eradicate the disease. To better understand the resistance phenotype, artemisinin-resistant parasite lines were derived from two clones of the 3D7 strain of *P*. *falciparum* using a selection regimen that mimics how parasites interact with the drug within patients. This long term *in vitro* selection induced profound stage-specific resistance to artemisinin and its relative compounds. Chemosensitivity and transcriptional profiling of artemisinin-resistant parasites indicate that enhanced adaptive responses against oxidative stress and protein damage are associated with decreased artemisinin susceptibility. This corroborates our previous findings implicating these cellular functions in artemisinin resistance in natural infections. Genomic characterization of the two derived parasite lines revealed a spectrum of sequence and copy number polymorphisms that could play a role in regulating artemisinin response, but did not include mutations in *pfk13*, the main marker of artemisinin resistance in Southeast Asia. Taken together, here we present a functional *in vitro* model of artemisinin resistance that is underlined by a new set of genetic polymorphisms as potential genetic markers.

## Author summary

The emergence of artemisinin resistance within and beyond Southeast Asia is a looming threat that needs to be promptly addressed. With this in mind, we derived several artemisinin-resistant parasite lines *in vitro* in order to fully characterize the resistance phenotype at the cellular and molecular levels. In addition to reinforcing the role of stress responses in mediating artemisinin resistance, we also identified novel genetic alterations that could also be responsible for causing artemisinin resistance. Collectively, this work provides additional insight in relation to, and beyond the paradigm of *pfk13*-driven artemisinin resistance and artemisinin response in *P*. *falciparum*. Understanding the processes that govern the acquisition of artemisinin resistance could aid in the development of strategies to prevent and contain it.

## Introduction

Malaria remains the most prevalent and deadly vector-borne disease in the world, with an estimated two hundred million cases and over four hundred thousand deaths recorded in 2015[1]. Currently, the cornerstone of global malaria control programs is artemisinin combination therapy (ACT). ACT combines the highly potent, rapidly acting artemisinin-based compounds with long-lasting partner drugs[2]. Artemisinin-based compounds have an excellent safety profile, exert a very rapid parasitocidal effect, and are active against gametocytes and all stages of the intraerythrocytic developmental cycle (IDC), from the early rings to the mature schizonts[3,4]. In particular, artemisinin compounds are typified by their short plasma elimination half-life, ranging from <1 to 3 hours for the water-soluble artesunate (ATS) and dihydroartemisinin (DHA), and from 3 to 11 hours for the oil-soluble artemether[3]. This is in sharp contrast to other antimalarial drugs that have considerably slower elimination times, persisting over several days to several weeks[5]. Hence, artemisinin-based drugs are the frontline therapies used by most, if not all, malaria control programs around the world.

In spite of its wide use, understanding of the artemisinin mode of action remains limited. Artemisinin belongs to the class of sesquiterpene lactones with an endoperoxide bridge that is essential for its antimalarial activity[6,7]. It is widely accepted that artemisinin-mediated parasite killing requires bioactivation of the peroxide structure that leads to generation of reactive oxygen species (ROS) and subsequent damage of biomolecules such as proteins, lipids and nucleic acids[6,7,8]. Some notable protein targets of artemisinin include: PfTCTP, a translationally controlled tumor protein homolog[9] which is located in both the cytoplasm and the food vacuole[10]; Pfatp6[11], an ER-resident, parasite ortholog of sarco/endoplasmic reticulum membrane calcium ATPase; and Pfpi3k[12], which is thought to be an early ring stage target of dihydroartemisinin. Artemisinin-derived radicals have been also shown to alkylate heme[13], which could lead to the disruption of hemozoin synthesis[14] which is essential to parasite survival. Additionally, this class of drugs have also been found to induce the ROS-mediated depolarization of both the mitochondrial[15,16] and plasma membranes[16], representing a different mechanism of parasite killing. It could very well be that the potency of endoperoxide-based drugs against the asexual blood stage is due to their ability to interact with a wide range of targets, across multiple cellular compartments[17,18]. However, the true impact of these interactions on parasite killing remains to be fully understood and requires further investigation.

Indeed, the use of artemisinin combination therapy has led to major progress in malaria control throughout the world in the last two decades, paving the way for better cure rates and reduced transmissibility in the field. From the late 2000s, however, pockets of decreased drug sensitivity to artemisinin-based drugs have been found in Southeast Asia. First detected in western Cambodia[19,20,21], resistance has been now reported from multiple locations across Asia including Thailand[22,23], Myanmar[23,24], Vietnam[23,25], and even Southern China[26]. It is believed that artemisinin resistance is continuously emerging *de novo*[27,28,29], but a few fit lineages are now spreading regionally[30]. What was originally characterized by delayed parasite clearance among patients treated with ACTs has now escalated to an alarming surge in treatment failures[31]. Interestingly, standard *ex vivo* 72-hour drug assays that are typically used to measure drug sensitivity are not able to differentiate between the slow-clearing (artemisinin-resistant) and fast-clearing (artemisinin-sensitive) parasites[32]. Instead, the Southeast Asian field isolates exhibit decreased susceptibility to artemisinins only in the very early ring stage of the IDC[32]. Transcriptional and cellular characterization of the resistant isolates demonstrated a delayed progression of the first half of the IDC, particularly the ring stage that is also the least susceptible to artemisinin[33,34,35]. These parasites are also characterized by upregulation of several cellular stress response pathways related to antioxidant defense and the unfolded protein response (UPR)[34]. Crucially, Ariey *et a*.*l* 2014 identified a biomarker of clinical artemisinin resistance that can be found in both *in vitro* and *in vivo P*. *falciparum* isolates[36]. After sequencing over 150 Cambodian isolates, they found several nonsynonymous single nucleotide polymorphisms (SNPs) in *pfk13* located at chromosome 13 that was similarly mutated in an artemisinin-resistant parasite derived *in vitro* by artemisinin exposure for over five years[36,37]. Interestingly, the chromosome 13 region around *pfk13* was identified independently as one of the genetic regions with a strong signature of selection among Thai and Cambodian parasites with slow clearance rates[38,39]. Subsequent surveillance of Southeast Asian isolates with different genetic backgrounds further corroborated *pfk13* as a strong genetic correlate of delayed parasite clearance[23,27,28]. Finally, functional studies validated that specific amino acid changes within the Pfk13 propeller domain significantly increases the rate of parasite survival after early ring-stage treatment with DHA[40]. Although *pfk13* is currently the best-characterized molecular marker, many questions remain about the mechanistic links between the amino acid changes in Pfk13 (a putative factor of intracellular protein-protein interactions) and the parasite’s resilience to artemisinin. It is particularly important to uncover all molecular components of the artemisinin resistance mechanism that can act in either a *pfk13*-dependent or -independent manner. Here we identified several putative factors that can facilitate artemisinin resistance by deriving and characterizing two artemisinin-resistant parasite lines from the *P*. *falciparum* 3D7 strain. Through the genomic, transcriptional and chemosensitivity profiling of these *in vitro* artemisinin-resistant parasites, our findings corroborate the central role of the parasite’s stress responses in mediating artemisinin resistance in *Plasmodium*, as well as demonstrate the possibility of a robust resistance phenotype that is potentially clinically relevant and is driven by different genomic alterations beyond *pfk13*.

## Results

### Selection of artemisinin resistant *P*. *falciparum* cell lines

**Fig 1:**
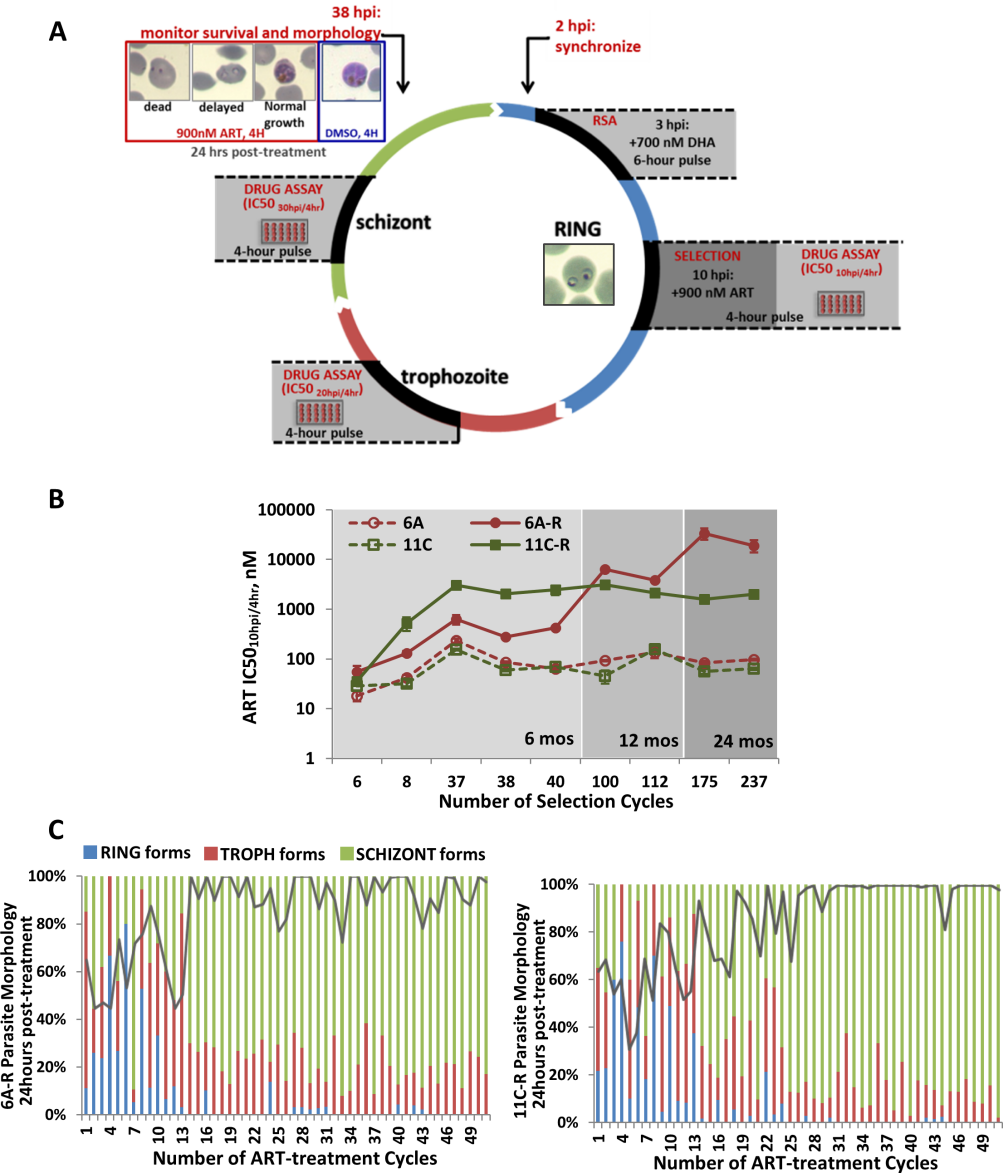
*In vitro* selection of artemisinin resistance in two *P*. *falciparum* clones 6A and 11C. Artemisinin resistance was induced in two subclones (6A and 11C) of the 3D7 strain of *P*. *falciparum* through periodic exposure of the parasite to short pulses of a clinically relevant dose of artemisinin. **(A)**The *in vitro* artemisinin selection protocol involved repeated 4-hour pulse treatments of synchronized mid-ring stage parasites (6A and 11C) to 900 nM artemisinin. DMSO-treated parasites were grown alongside the artemisinin-treated parasites (renamed as 6A-R and 11C-R) to serve as controls. Both sets of parasites were subjected to the same number of artemisinin and DMSO treatments throughout drug selection, and at the same generations. Stage-specific artemisinin sensitivity was monitored throughout the course of selection using a 4-hour drug pulse assay at the ring (IC50_10hpi/4hr_), trophozoite (IC50_20hpi/4hr_), and schizont (IC50_30hpi/4hr_) stages of the IDC. **(B)**In order to monitor incremental changes in ring stage artemisinin sensitivity over time, artemisinin IC50_10hpi/4hr_ was measured throughout increasing cycles of drug selection between artemisinin-treated parasites and their controls. Additional data can be found in **S1 Table**. **(C)**At the start of artemisinin selection, parasite viability and morphology after 4-hour treatment was monitored using microscopic evaluation of Giemsa-stained blood smears. The solid gray line depicts the proportion of surviving parasites 24 hours post treatment normalized to the starting parasitemia, while stacked bars depict proportions of ring, trophozoite and schizont stage morphologies observed among the remaining parasites that appeared to be viable. Examples of parasite morphologies after pulse artemisinin treatment are depicted in **S1 Fig**.

The overall goal of this research was to identify and characterize molecular factors that contribute to resistance of the malaria parasite *P*. *falciparum*, to artemisinin. For this purpose, we derived two parasite lines from two isogenic clones of the 3D7 strain termed 6A and 11C[41]. This was done by repeated exposures of synchronized parasite cultures to 900 nM of artemisinin for 4 hours at the ring stage (10–14 hours post invasion, HPI) (**Fig 1A**). At the initial earlier stages of the selection process, these pulse treatments were applied every other round of the IDC (see materials and methods). The main rationale of this selection regimen was to approximate clinical conditions in the peripheral blood of infected patients where artemisinin peaks at ~900nM[42] and decays below clinical levels within 2 to 5 hours[3], and where the *P*. *falciparum* populations consists predominantly of ring-stage parasites (~10 HPI)[33,43]. The ring stage that is otherwise the least sensitive to artemisinin[44], is believed to be driving the currently occurring artemisinin resistance phenotypes observed in natural infections[32,45]. Initially, during the first 13 treatment cycles, the rate of parasite survival after each artemisinin exposure fluctuated between 30–90% (**Fig 1C**). These surviving parasites were typically arrested in the ring/trophozoite stages for up to 24 hours post treatment instead of progressing to the expected schizont stages (**Fig 1A and 1C, S1 Fig**). However, from 18 cycles onwards, 70–100% of parasites were consistently surviving the treatment, progressing normally through the IDC (**Fig 1C**). We observed a marked decrease in artemisinin susceptibility in both clones as early as 6 rounds of treatment (26 days) for 6A-R, and 8 rounds of treatment for 11C-R (33 days). At that stage the 6A-R and 11C-R exhibited a 3- and 17-fold increase of artemisinin resistance, respectively, as measured by a survival assay establishing the 50% inhibition concentration for parasites exposed to the drug for 4 hours at 10 HPI (IC50_10hpi/4hr_) (**Fig 1B, S1 Table**). This drug pulse assay was designed to match the window of the drug selection, resembling the previously utilized shorter exposure drug assays that were shown to capture the stage-dependent artemisinin activities[44,46]. Using this assay, we were observed marked differences in the dynamics of the progression of artemisinin resistance between the two clones throughout the drug selection regimen (**Fig 1B, S1 Table**). 6A-R showed a gradual increase of IC50_10hpi/4hr_, starting at 55.49 nM at 6 cycles of artemisinin exposures, progressing to 3,880 nM after 11 months and peaking at 33,726 nM after approximately 1.5 years of continuous treatments. On the other hand, 11C-R exhibited a rapid increase of resistance between 6 and 37 cycles (first 5 months of drug selection) to IC50_10hpi/4hr_ = 3,052 nM. Subsequently, this level of resistance plateaued for the next 19 months of continuous cultivation under drug selection (**Fig 1B**). Hence, compared to their corresponding parental lines, the resulting artemisinin resistant lines 6A-R and 11C-R exhibited up to a 398- and 69-fold increase in IC50_10hpi/4hr_, respectively. The drug resistance phenotypes of both lines remained fully intact in parasites that were cryopreserved and reintroduced to culture. Moreover, a significantly elevated IC50_10hpi/4hr_ was maintained after three months of cultivation in the complete absence of drug pressure. In summary, here we derived two artemisinin resistant lines of *P*. *falciparum* that could be actively maintained in an *in vitro* culture and thus serve as a tool for mechanistic studies of artemisinin resistance. The differences in the resistance levels and selection dynamics suggest that the two resistant parasite lines employ (to at least some degree) distinct molecular factors to withstand artemisinin.

### Drug resistance phenotypes

**Fig 2:**
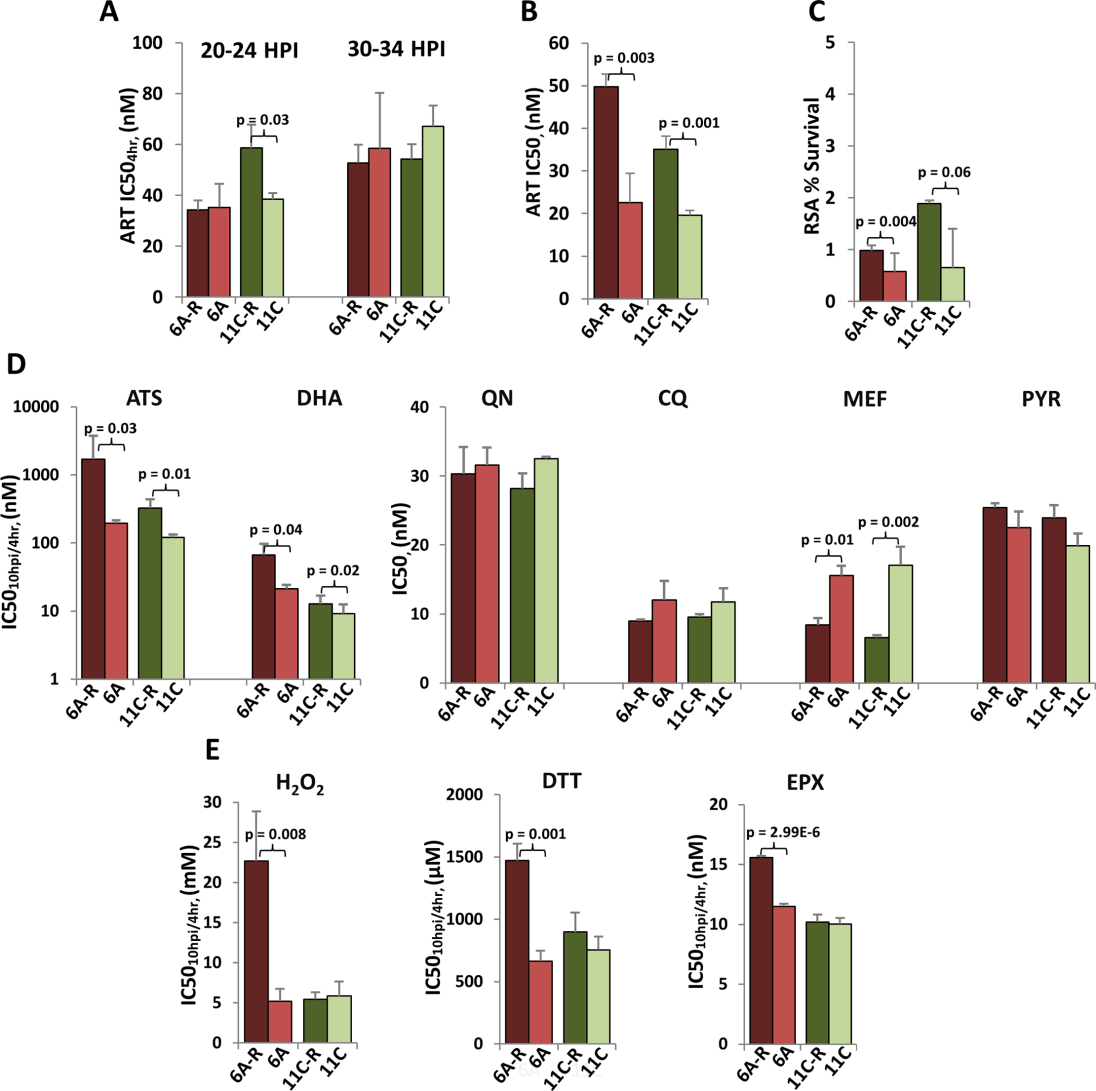
Chemosensitivity profiling of *in vitro*-selected *P*. *falciparum*. Chemosensitivity phenotypes of our artemisinin-resistant parasites were evaluated. **(A)**Apart from their ring-stage sensitivity, the artemisinin IC50_4hr_ for the trophozoite (IC50_20hpi/4hr_) and schizont (IC50_30hpi/4hr_) stages was also measured in all parasite lines. Differential susceptibility to artemisinin between selected and control parasites, was subsequently validated using a standard *in vitro* (72-hour) drug assay (IC50) **(B)**, and the Ring stage Survival Assay (RSA) **(C)**. Additional data can be found in **Figs 2A and S2B** and **S2 Table**. **(D)** Ring-stage susceptibility (IC50_10hpi/4hr_) against a pulse exposure to artemisinin derivatives Dihydroartemisinin (DHA) and Artesunate (ATS) were also monitored, while chemosensitivity against non-artemisinin antimalarials, quinine (QN), chloroquine (CQ), mefloquine (MEF) and pyrimethamine (PYR), were evaluated using a standard drug assay (IC50). Additional data can be found in **S3A and S3B Fig** and **S3 Table**. **(E)**Apart from antimalarial drugs, the ring-stage sensitivity of all parasite lines was also measured for compounds that are related to the mechanism of action of artemisinin: hydrogen peroxide (H_2_O_2_), dithiothreitol (DTT), and epoxomicin (EPX). Additional data can be found in **S3C Fig** and **S3 Table**. All drug assays were performed in biological triplicates; error bars represent the standard deviation. Pairwise comparison of percent survival (RSA), IC50_10hpi/4hr_ and IC50 values between resistant and sensitive parasites was performed using student’s t-test.

Interestingly, the derived resistance phenotype(s) of both 6A-R and 11C-R are predominant in the rings (10 HPI) and do not affect the later stages of IDC development (**Fig 2A, S2A Fig, S2 Table**). However, for 11C-R, the window of resistance extends until the early trophozoite stage (~20 HPI), where a moderate level of resistance can still be observed. The robustness of the ring-specific artemisinin resistance is likely the main reason for the observed resistance demonstrated by both parasite lines in the standard 72-hour drug assay that measures parasite survival after artemisinin exposure across all stages of the IDC **(Fig 2B, S2B Fig, S2 Table)**. Crucially, both parasite lines also showed decreased drug susceptibility in the ring survival assay (RSA)[32,45] carried out with parasites at 0–3 HPI (**Fig 2C**). Both 6A-R and 11C-R passed the 1% RSA survival cutoff employed in the field to denote resistance[45,47,48]. This contrasts the current phenotype observed in natural infections that exhibit high levels of ring-stage specific resistance (in the RSA), but show no changes in the standard 72-hour drug assay[32]. Both 6A-R and 11C-R also have significantly elevated IC50_10hpi/4hr_ to other semisynthetic artemisinin derivatives. 6A-R exhibited 5- and 8-fold higher IC50_10hpi/4hr_ to dihydroartemisinin (DHA) and artesunate (ATS), and 11C-R showed 2- and 3-fold higher IC50_10hpi/4hr_ to DHA and ATS, respectively. Both, 6A-R and 11C-R, however, showed no changes in sensitivities to other antimalarial drugs including two quinolines (quinine and chloroquine) and pyrimethamine (**Fig 2D, S3A and S3B Fig, S3 Table**). Taken together these results suggest that the derived resistance phenotypes are specific to artemisinin and its endoperoxide-carrying derivatives, and can give rise to full resistance phenotypes of the *P*. *falciparum* parasites. Given its relevance in the RSA, these mechanisms may correspond to the current artemisinin resistance in natural infections albeit being independent from *pfK13* polymorphisms[32,36] (see below). Interestingly, both parasite lines exhibited increased susceptibility to mefloquine, whose mode of action is presumably related to other quinolines[49]. In future studies, it will be interesting to investigate the relationship between the altered sensitivities of *P*. *falciparum* to artemisinins and mefloquine. However, here it is important to note that the connection between these two chemosensitivity phenotypes is not absolute as observed in another artemisinin-resistant line derived from a polyclonal population of the T996 *P*. *falciparum* strain (**S3D Fig**).

Stress responses to an oxidative damage and the unfolded protein response (UPR) have been implicated in the mechanisms of artemisinin resistance of *P*. *falciparum* in *in vitro* cultures [50,51] and natural infections[34]. To investigate the role of these two biological processes in the derived resistant parasite lines, we challenged our *in vitro*-derived resistant parasites with H_2_O_2_, dithiothreitol (DTT) and epoxomicin (EPX). While H_2_O_2_ causes oxidative damage, DTT and EPX are inducers of ER stress, causing an accumulation of damaged/misfolded proteins inside the cell. Intriguingly, 6A-R, but not 11C-R, exhibited a significant resistance to all three inhibitors (**Fig 2E, S3C Fig, S3 Table**). This is consistent with our previous suggestion of inherent mechanistic differences in the artemisinin resistance mechanisms between 6A-R and 11C-R and shows that oxidative damage repair and unfolded protein responses play a central role in artemisinin resistance as observed *in vivo* [33,34,51].

### Transcriptomic profiling of artemisinin-resistant lines

**Fig 3:**
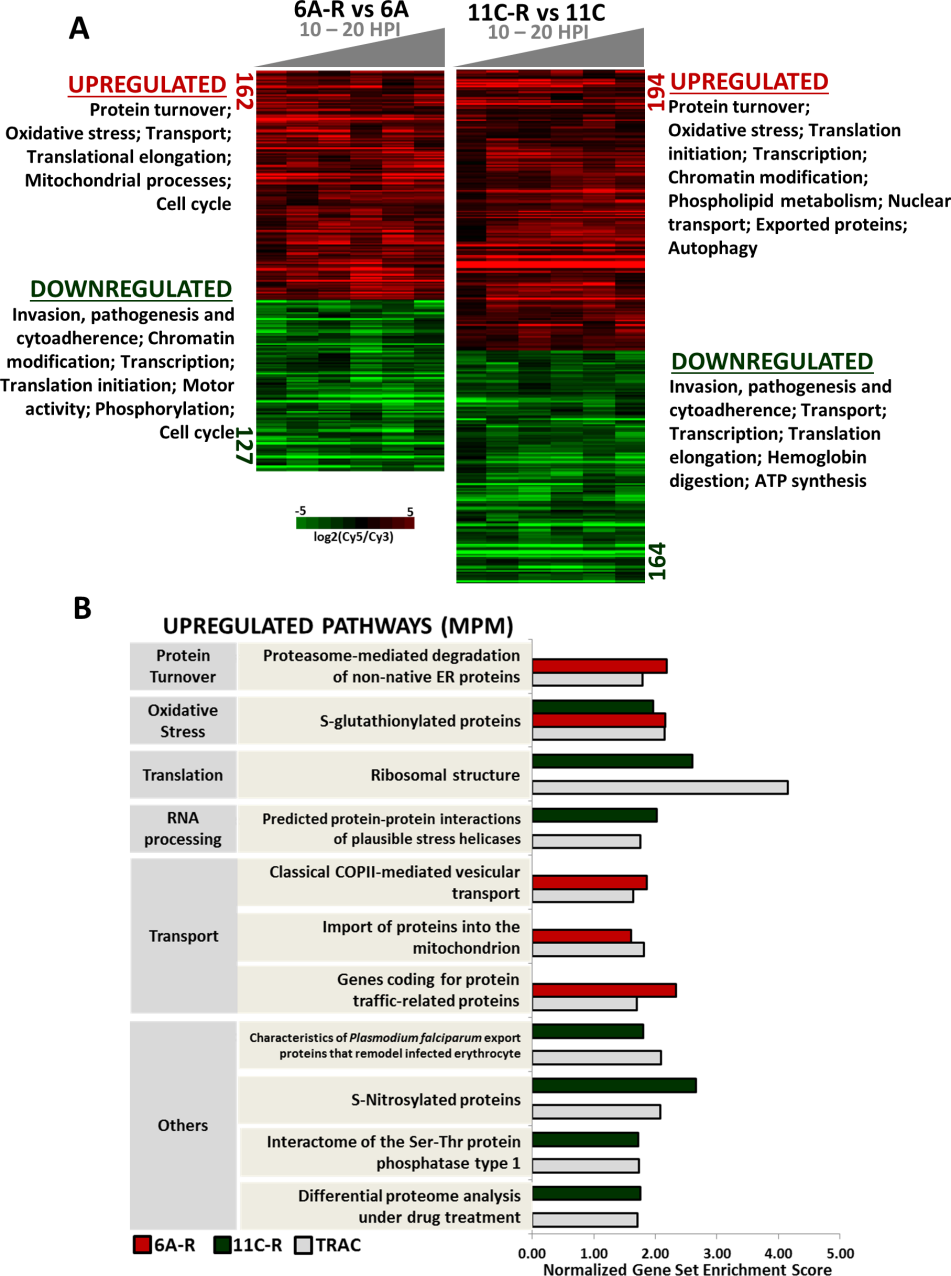
Transcriptional profiling of *in vitro*-selected *P*. *falciparum*. Comparative transcriptomic analysis was also done on resistant vs sensitive parasites across the mid to late ring stage (10–20 HPI), which matches the window of drug treatment. (A)Heatmaps depict significantly up- and downregulated genes (corrected p-value < 0.05, FDR < 0.25) in artemisinin-resistant parasites relative to their artemisinin-sensitive controls. Noted are the differentially expressed pathways between artemisinin-resistant and artemisinin-sensitive parasites identified using Gene Set Enrichment Analysis (GSEA) (p < 0.05, FDR < 0.25). All genes from the total transcriptomic datasets were ranked by their z-score based on the difference in expression between resistant and sensitive lines, and subsequently subjected to GSEA. A more comprehensive list of differentially expressed pathways and genes between resistant and sensitive parasite lines can be found in Table A in S4 Table and S1A File (6A-R vs 6A), and Table B in S4 Table and S1B File (11C-R vs 11C). Data shown represents time-course transcriptomes over a single IDC. (B)The graph depicts the overlap between significantly upregulated functionalities (GSEA p-value < 0.05, FDR < 0.25) in ring-stage artemisinin-resistant clinical isolates (from TRACI) and our *in vitro*-selected artemisinin resistant parasites. All 5,061 genes from the previously published TRAC I dataset[34], were ranked by their strength of association with parasite clearance half-life (correlation coefficient) and used for GSEA.

To assess whether the *in vitro*-derived artemisinin resistant phenotypes reflect the similar physiological state observed *in vivo*, we characterized the transcriptomes of 6A-R and 11C-R. First we reconstruct the IDC transcriptomes of both resistant clones grown under normal conditions (**S4A Fig**). The “best fit” parasite aging analysis[33] showed that starting from the mid ring stage (time point 2), both lines progressed identically and completed their IDC in approximately 48 hours. However, both resistant parasite lines appeared to accelerate their early ring stage progression being older (10 HPI) than their sensitive counterparts (4 HPI) at the first sampling interval (**S4A Fig**). This observation is consistent with the ring-specific resistance in both clones and their resistance in the RSA that appear to be involved in the *pfk13*-dependent artemisinin resistance observed *in vivo*. Examining the transcriptomes of 6A-R and 11C-R between 10–20 HPI, we detected broad alterations in mRNA levels of >300 *P*. *falciparum* genes (corrected p-value < 0.05, FDR < 0.25), as well as changes in key processes that might be linked to modulating artemisinin response in the parasite (**Fig 3A**). In 6A-R, pathways related to the redox stress responses and protein turnover were predominant amongst the upregulated genes. Notably, we observed an upregulation of genes that may be related to the parasite’s thioredoxin-based redox system such PF3D7_1457200 (thioredoxin 1), PF3D7_1438900 (thioredoxin peroxidase 1), and PF3D7_1352500 (thioredoxin-related protein). We also observed an enrichment of targets of glutathionylation, as well as targets of the thioredoxin enzyme superfamily. The upregulated protein turnover-associated genes included heat shock and chaperone proteins, and a number of enzymes involved in proteolysis. We also observed an upregulation of genes involved in translational elongation, electron transport, and protein transport, particularly vesicular trafficking between the ER and Golgi complex. On the other hand, the significantly downregulated genes were enriched for pathways related to host-parasite interactions, control of gene expression, and translational initiation (**Fig 3A, Table A in S4 Table and S1A File**). Interestingly, gene sets involved in cell cycle regulation were also found to be differentially expressed between 6A-R and 6A—which could be related to the slight shift in temporal progression during the early stages of parasite development. In the case of 11C-R, we likewise observed a significant upregulation of genes involved in oxidative stress defense, although to a lesser extent compared to 6A-R. These include genes that encode S-glutathionylated proteins, PF3D7_0306300 (glutaredoxin 1) and PF3D7_0709200 (glutaredoxin-like protein). Pathways involved in protein damage repair, including chaperones and components of proteasome-mediated degradation are also overexpressed. In addition, 11C-R exhibited an upregulation of processes related to early translation events, and transcriptional and post-transcriptional mechanisms of gene regulation such as chromatin modification, stress helicase activity, and the formation of P-bodies. Induction of P-bodies has been observed under stress or conditions that repress translation initiation[52,53,54], and their role in drug resistance may not be ruled out. As for the significantly downregulated functionalities in 11C-R, we identified factors of host-parasite interactions, components of the transcriptional machinery, cellular transport, hemoglobin digestion, several translational elongation factors and ATP synthesis (**Fig 3A, Table B in S4 Table and S1B File)**. Evaluating the transcriptional correspondence of 6A-R and 11C-R with slow clearing isolates in Southeast Asia from the TRAC I[34], we found a great degree of overlap between significantly upregulated pathways in the *in vitro* and *in vivo* datasets (**Fig 3B**). Strikingly, several of these pathways have also been associated with longer parasite clearance half-lives in the field such as coping mechanisms against ER stress (ER trafficking, proteasome-mediated degradation, translation) and oxidative stress (targets of glutathionylation), as well as mRNA processing[34]. Not only does this observation reinforce the involvement of these cellular processes in modulating artemisinin resistance in *Plasmodium*, it also demonstrates that 6A-R and 11C-R are each able to recapitulate key aspects of *in vivo* artemisinin resistance at the transcriptional level.

Next we analyzed global transcriptional responses of 6A-R and 11C-R to artemisinin drug exposure that is identical to the selection conditions (synchronized parasites were treated with 900nM artemisinin from 10 to 14 HPI) (**S4B Fig**). Here we observed many similarities between how 6A-R and 11C-R respond to a ring-stage artemisinin challenge in relation to their sensitive counterparts. Notably, both lines exhibit a downregulation of processes pertaining to pathogenesis, transcriptional control, translation, cellular transport and cell cycle regulation (**S4B Fig, Tables A and B in S5 Table**). That both *in vitro*-derived lines demonstrate a marked dysregulation of genes involved in cell cycle regulation could be related to their ability to overcome the drug induced quiescence caused by artemisinin. Interestingly, GSEA identified transport across the ER-Golgi and digestive vacuole (DV) membranes as significantly upregulated in 11C-R (**S4B Fig, Table B in S5 Table and S2B File)**. Likewise, 6A-R also displayed an upregulation in transmembrane transport components—a number of which have been linked to drug resistance in *Plasmodium*. A notable example is the DV-resident chloroquine resistance transporter, *pfcrt*, which is significantly upregulated in both resistant lines and has been associated with chloroquine resistance [55,56,57,58]. *Pfcrt* also plays a role in glutathione transport and antioxidant defense within the DV[59]. *Pfexp1*, a glutathione transferase located on the parasitophorous vacuole and is associated with artesunate sensitivity and metabolism[60] is also found to be upregulated in 6A-R (**S2A File**). On the other hand, we also observed transcriptional features that are distinct to only one parasite line, such as the downregulation of autophagy-related pathways in 6A-R vs. 6A, and the observed downregulation of heat shock proteins in 11C-R compared the 11C. It is probable that the differences we observed in global transcriptional profiles between 6A-R and 11C-R could account for some of the phenotypic variations between these two parasite lines such as resistance to H_2_O_2_/DTT/EPX. In the future it will be interesting to study these variations as they could represent genuine differences in drug resistance phenotypes *in vivo*.

### Genomic characterizations of artemisinin-resistant lines

The whole genome sequencing of the two resistant parasite lines identified several intragenic SNPs compared to their parental lines. These included 3 and 5 missense mutations in 6A-R and 11C-R, respectively; one nonsense mutation in each parasite line and an additional intronic SNP in 6A-R (**Table 1**). As a result, there are mutation alleles for five genes in 6A-R and six genes in 11C-R. Cross-referencing our SNP data with the Pf3k[61] and MalariaGEN[62] databases, the nonsynonymous mutations detected in PF3D7_1427100, PF3D7_0810600 and PF3D7_1115700 were found to also occur in natural infections of African origin. Crucially, there was no overlap between the mutated genes in the two parasite lines, both of which also carried the wild-type allele of the K13 gene (validated by PCR-based genotyping of *pfk13*[36,63]). No polymorphisms were also detected in previously identified drug resistance markers, such as *pfcrt*[56,64], *pfmrp1*[65,66], *pfmdr1*[67], *pfnhe-1*[68], *pfdhps*[69], *pfdhfr*[70,71], *pfatp6*[72], *pfubp1*[73], *pfap2mu*[74] PF3D7_101700[39], and PF3D7_1343400[39]. Moreover, none of the SNP-containing genes in 6A-R and 11C-R match the previously reported putative targets and interacting partners of artemisinin, such as *pfatp6*[11], *pfpi3k*[12], *pftctcp*[9] and other proteins [17,18]. The only exception is the nonsense mutation in *pffp2a* (PF3D7_1115700) that encodes falcipain 2a, the main factor of hemoglobin digestion, whose nonsense polymorphism was previously linked with artemisinin resistance *in vitro*[36,37]. On the other hand, both 6A-R and 11C-R harbor mutations in genes that might play a role in gene expression regulation such as AP2-like transcription factors, a PHD finger protein and an RNA helicase. Such genes could be implicated in the regulation of the *Plasmodium* IDC transcriptional cascade and subsequently contribute to the resistance phenotypes of both parasite lines.

**Table 1:** Single nucleotide polymorphisms (SNPs) identified in 6A-R and 11C-R.

	GENE ID	DESCRIPTION	SNP	EFFECT
**6A-R**	**PF3D7_0704800**	conserved Plasmodium protein, unknown	T241086C	T322V
	**PF3D7_0730300**	transcription factor with AP2 domain(s)	T1301352G	H1298Q
	**PF3D7_1115700**	cysteine proteinase falcipain 2a	G593481T	S35stop
	**PF3D7_1141800**	phd finger protein, putative	A1673564G	intron
	**PF3D7_1427100**	lipase, putative	G1059278T	W1039C
**11C-R**	**PF3D7_0420300**	transcription factor with AP2 domain(s)	A923966G	T1993A
	**PF3D7_0528300**	conserved Plasmodium protein, unknown	G1167265T	P249H
	**PF3D7_0617100**	alpha adaptin-like protein, putative	A712508C	H817P
	**PF3D7_0810600**	RNA helicase, putative	G543210T	A414S
	**PF3D7_1230000**	conserved Plasmodium protein, unknown	C1237510A	V815stop
	**PF3D7_1368400**	conserved Plasmodium protein, unknown	A2720248T	F202Y

All artemisinin-resistant and artemisinin-sensitive lines were subjected to high throughput sequencing using the Illumina MiSeq platform, and nonsynonymous intragenic single nucleotide polymorphisms (SNPs) were called using SAMtools. Only high quality SNPs with at least 75% position coverage, and not present in control parasite lines are presented in this table. Mutations in the hypervariable multigene families were excluded from the analysis.

**Fig 4:**
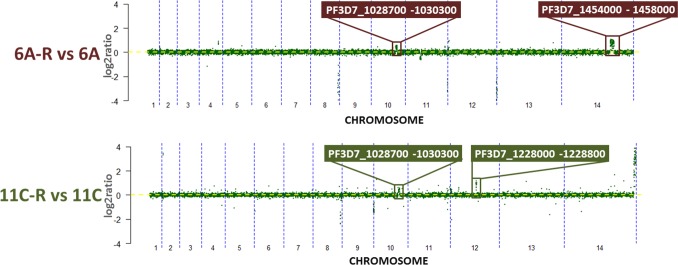
CNV profiling of *in vitro-*selected *P*. *falciparum*. Copy number variations in 6A-R and 11C-R were identified using microarray-based comparative genomic hybridization (CGH). Chromosome plots reflect the subtracted log_2_ratio of the artemisinin-resistant parasite lines relative to their control counterparts. Copy number variable genes in 6A-R vs. 6A, and 11C-R vs 11C are indicated in the red and green boxes, respectively. Additional data can be found in **S5 Fig** and **S6 Table**.

Next, we characterized the genome-wide patterns of copy number variations (CNVs) using microarray-based comparative genomic hybridization (CGH) as previously described[41]. In both 6A-R and 11C-R, we detected two gDNA segments whose amplifications could be directly related to their artemisinin resistance status (**Fig 4, S6 Table**). Namely, there is a segment on chromosome 14 spanning 40 genes (PF3D7_1454000-PF3D7_1458000) amplified in 6A-R, and a segment on chromosome 12 spanning 9 genes (PF3D7_1228000—PF3D7_1228800) amplified in 11C-R. Moreover, both 6A-R and 11C-R also carry a common amplification on chromosome 10 spanning 17 genes (PF3D7_1028700—PF3D7_1030300). This selfsame amplification has also been identified previously in an artemisinin sensitive *P*. *falciparum* 3D7[75] strain. The three CNVs on chromosomes 10, 12 and 14 were detected during the later stages of drug selection and subsequent culturing, and were consistently detected over a period of five months (89 generations) (**S5 Fig**). Comparing our CNVs with a dataset collated from 122 clinical isolates from Africa, South East Asia and South America[76], we found that none of the isolates contained the chromosome 10, chromosome 12 and chromosome 14 amplification clusters in their entirety. However, one isolate collected from Peru harbored a copy gain for the putative gamma-adaptin encoding PF3D7_1455500.

### Impact of overexpression of stress response genes on artemisinin resistance

**Fig 5:**
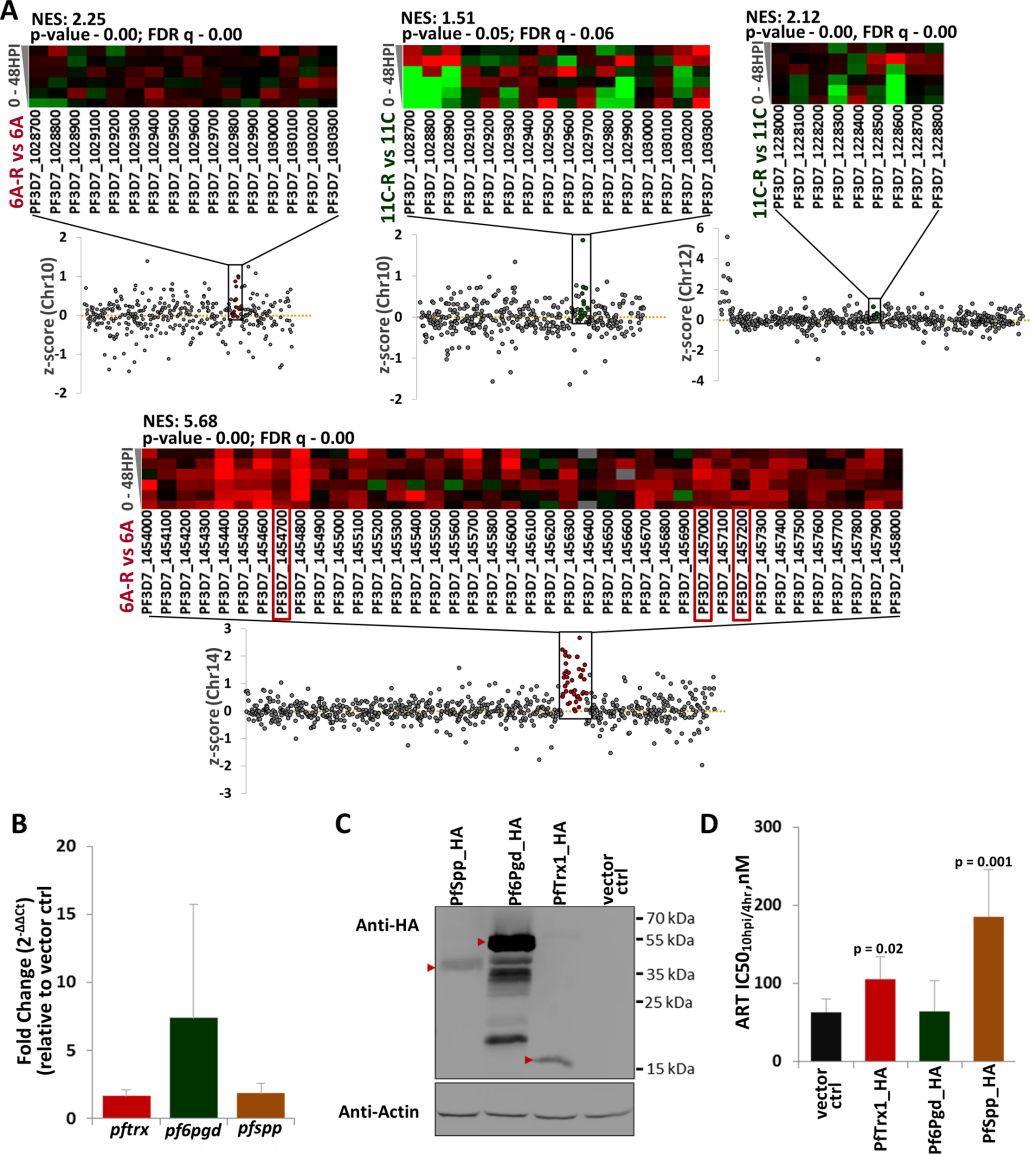
Effect of overexpression of *pftrx1*, *pf6Pgd* and *pfspp* on artemisinin sensitivity. To validate the role of stress response gene overexpression on artemisinin resistance, transfectant lines overexpressing select candidate genes were generated, and subsequently assayed for artemisinin sensitivity. **(A)**Differential mRNA expression in the copy number-amplified genes identified in chromosomes in 10, 12, and 14 was evaluated between artemisinin-resistant parasite lines and their corresponding controls. **S6 Table** lists down corrected p- and FDR values for each gene in the Chr 10, Chr 12 and Chr 14 CNV clusters identified. Chromosome plots depict the z-score calculated for each gene based on differences in expression levels between resistant and sensitive parasites across the IDC, while the heatmaps represent the fold-difference between resistant and sensitive parasites for each gene at 6 timepoints taken at 8-hour intervals across a single IDC. Also indicated are the Normalized Enrichment Score (NES) values for transcriptional upregulation in each cluster, obtained by GSEA (p-value < 0.05, FDR < 0.25). Marked in red boxes are candidate stress response genes that were found to be significantly upregulated across the IDC (corrected p-value < 0.05, FDR < 0.25) and amplified in 6A-R. These three genes (PF3D7_1454700 (*pf6pgd*), PF3D7_1457000 (*pfspp)*, and PF3D7_1457200 (*pftrx1*)) were subsequently episomally overexpressed and investigated for their capacity to modulate artemisinin sensitivity. Prior to phenotyping, **(B)**Real-time qPCR was used to determine the relative overexpression of *pftrx1*, *pf6pgd* and *pfspp* from their respective overexpression parasite lines. Mean fold-change values are derived from three biological replicates; error bars represent the standard deviation. **(C)**Western blot analysis was also carried out on all overexpression and control parasite lines using a monoclonal mouse anti-HA antibody to validate tagged-protein production. Bands denoted by red arrows indicate the tagged proteins at their expected molecular weights. **(D)**Ring-stage artemisinin sensitivity (IC50_10hpi/4hr_) was then measured for all overexpression parasite lines and compared against the vector control. Drug assays were performed in biological triplicates; error bars represent the standard deviation. Pairwise comparison of IC50_10hpi/4hr_ between each overexpression line and the vector control was performed using student’s t-test. Additional data can be found in **S6** Fig and **S7 Table**.

Given the scope of the detected transcriptional changes in 6A-R and 11C-R, we wished to investigate the possibility that CNV-driven variations in expression can mediate artemisinin resistance. Evaluating the individual expression levels of each gene in the three CNV clusters identified, we found that not all transcripts appear to be significantly overexpressed across the IDC between resistant parasites and their sensitive counterparts (**Fig 5A and S6 Table**). However, comparing the collective expression among the amplified genes on chromosomes 10, 12 and 14, we were able to detect a significant enrichment of upregulation in the genes located in these regions (**Fig 5A**). This observation is particularly striking in the case of the chromosome 14 amplification in 6A-R, where 30 out of the 40 genes were significantly overexpressed across the IDC (**S6 Table**). Here we focused on three genes on the 6A-R chromosome 14 amplification that are likely to be involved in adaptive responses against cellular damage within the parasite. These include 6-phosphogluconate dehydrogenase (PF3D7_1454700, *pf6pgd*) and thioredoxin 1 (PF3D7_1457200, *pftrx1*)—both of which are involved in antioxidant defense[77,78,79], and an ER-resident signal peptide peptidase (PF3D7_1457000, *pfspp)*[80] that is a component of ER associated degradation (ERAD)[81]. All three candidate genes were found to be significantly overexpressed in 6A-R compared throughout the IDC (**Fig 5A and S6 Table**). In order to assess their potential to confer artemisinin resistance, we generated transgenic *P*. *falciparum* lines in which each candidate gene was overexpressed episomally. Briefly, each gene was fused with the HA-antibody epitope at the C-terminus and cloned into the pBcamR_3xHA transfection vector (see materials and methods) that allows adjustable expression via increased copy number driven by blasticidin (BSD). Quantitative RT-PCR demonstrated increased transcription of the transgenic contracts by 7-fold for *pf6pgd* and 2-3-fold for *pftrx1* and *pfspp* (**Fig 5B**). Western blot analysis confirmed the production of the HA-tagged transgene protein products at their expected molecular weights in the transgenic cell lines grown in the presence of 2.5 ug/mL BSD (**Fig 5C**). Crucially, overexpression of *pftrx1*, and *pfspp* resulted in a subtle but significant decrease in artemisinin sensitivity, with IC50_10hpi/4hr_ 1.7-fold, and 2.9-fold higher than the “empty vector” control, respectively (**Fig 5D, S6 Fig and S7 Table**). On the other hand, no significant difference in artemisinin sensitivity could be observed in the parasites overexpressing *pf6pgd*. These results indicate that the specific upregulation, possibly as a result of gene amplification, of *pftrx1* and *pfspp* contributed to the decreased sensitivity of 6A-R to artemisinin.

## Discussion

It has been previously shown that resistance can be induced in culture-adapted *P*. *falciparum* parasites through long-term exposures to artemisinin and/or its derivatives[37,50,82,83]. That includes the studies that discovered the current principal marker of artemisinin resistance in Southeast Asia, *pfk13* [36,37]. The identification of the *pfk13* gene highlights the value of such *in vitro* models to systematically investigate the mechanisms that drive artemisinin response and resistance in the clinical setting. Here, we developed two artemisinin resistant cell lines from isogenic clones of the 3D7 *P*. *falciparum* strain. For this study, we chose two isogenic clones of the 3D7 reference strain that has been previously extensively characterized, and thus will lead to efficient identification of all derived genetic variation. The 3D7 strains also represent a fully artemisin-susceptible background which provides a “naïve” baseline genome that potentially allows for the identification of causative factors of artemisinin resistance that are independent of any potential genetic background with a propensity for drug resistance[27,84]. This yielded a resistance phenotype(s) that is (are) distinct from the previous reports. Essentially all previously derived *P*. *falciparum* parasites involved an artemisinin-induced growth arrest and recovery as a major component of the resistance phenotype[37],[50],[85],[83]. In contrast, 6A-R and 11C-R are both characterized by an increased survival in the presence of artemisinin with no detectable levels of growth retardation or arrest. This marked difference is likely due to the pulse-based regimen that contrasts the previous studies in which the parasite lines were treated for considerably longer time periods, ranging from 24–48 hour drug exposure intervals[36,37] to continuous drug pressure[50,82,83]. Moreover, 6A-R and 11C-R displayed significant decreases of artemisinin sensitivity (IC50_10hpi/4hr_) within as early as 1.5 months of selection. This is also in stark contrast with previous reports by Witkowski *et al*. that showed that the chemosensitivity of the *P*. *falciparum* F32 strain remained unaltered for up to 3 years and/or 100 cycles of drug pressure when the parasites were treated with artemisinin for 24 hours at a time[37]. Similarly, Cui *et al*. were unable to raise drug resistance in the 3D7 strain at all and could only generate resistant parasites using other culture adapted *P*. *falciparum* strains including 7G8, Dd2, HB3 and D10 after at least one to two months of continuous exposure to DHA[50]. This collectively indicates that artemisinin resistance of *P*. *falciparum* could be derived by multiple ways, each of which may induce a distinct mechanism.

Unsurprisingly, the artemisinin resistance in both parasite lines extends to its cognate drugs ATS and DHA. But while 6A-R and 11C-R showed up to almost 400- and 70-fold increases in IC50_10hpi/4hr_ values for artemisinin (**Fig 1B, S1 Table**), respectively, both lines exhibited increases in IC50_10hpi/4hr_ for ATS and DHA by less than 10-fold (**Fig 2D, S3 Table**). This is likely a reflection of key differences in pharmacodynamic profiles between artemisinin and its two synthetic derivatives. Compared to the plant-derived artemisinin, both ATS and DHA are more potent antimalarials with DHA being the primary cytopathic metabolite responsible for the parasite killing[6,86]. In contrast, artemisinin is not metabolized to DHA but instead acts as the primary antimalarial agent itself and is subsequently transformed into inactive deoxyartemisinin and dihydrodeoxyartemisinin[4,87,88]. The variance in the resistance level of the two derived clones could be attributed to differences in the overall levels of the cytopathic activities, the mode of activation, and/or the protein targets that each compound is specifically engaging. Moreover, while 6A-R and 11C-R did not exhibit cross-resistance to other types of antimalarials, both clones are more susceptible to mefloquine (**Fig 2D**). Interestingly, DHA-resistant parasites previously derived by Cui *et al*. from a Dd2 parent, displayed decreased sensitivity to other artemisinin-based drugs albeit to a lesser extent compared to DHA, but also to quinine, chloroquine and mefloquine[50]. Furthermore, parasites derived using long term exposure to artelinic acid from the D6 and W2 backgrounds showed cross-resistance to mefloquine but increased susceptibility to chloroquine[82,83]. These findings allude to the possibility that resistance to artemisinin-based drugs could also affect the clinical efficacy of its partner drugs used in the currently deployed ACTs. These results highlight the importance of testing for cross-resistance as an integral part of drug development, and also demonstrate a key use for *in vitro* drug resistance models that can be utilized as a platform with which to perform such extensive and rigorous studies.

The *P*. *falciparum* parasites causing the current state of slow clearing infections in the Southeast Asian patients show are marked by higher RSA values[45] but show no differential sensitivities in standard *in vitro* drug assays[20,32,89]. These *in vivo* parasites are characterized by transcriptional induction of oxidative and (other types of) protein damage responses, and at the same time, a deceleration of the early stages the IDC [33,34]. Both of these transcriptional phenotypes are strongly linked with mutations in the *pfk13* gene as the main marker of artemisinin resistance [27,28,36]. Here we observed several main similarities between *in vivo* artemisinin resistance and the *in vitro-*derived phenotypes of 6A-R and 11C-R. First, like the slow-clearing isolates in Southeast Asia, the resistance of 6A-R and 11C-R is tied to the earlier stages of the IDC, and fades as the parasites progress into the later stages. This is reflected by an elevation in the RSA index (>1%) for both 6A-R and 11C-R that is comparable to the *in vivo* isolates (**Fig 2C**). Second, both resistant lines demonstrated a steady state upregulation of genes and pathways that are involved in antioxidant defense, as well as the UPR (**Fig 3A and 3B**)[34]. Crucially, the induced artemisinin resistance in the 6A-R clone also gave rise to cross-resistance against oxidative agents (e.g. H_2_O_2_), protein-folding disruptors (e.g. DTT), and stressors of protein processing in the ER (EPX) (**Fig 2E**). This indicates that that its derived resilience to artemisinin is tied to an increased capacity to mediate oxidative stress and protein damage. These findings suggest that one possible mechanism for artemisinin resistance is an enhanced ability of the *P*. *falciparum* parasites to cope with the oxidative stress and protein damage presumably caused by artemisinin directly. On the other hand, the *in vitro*-derived lines were unable to recapitulate certain features of the resistant isolates. Neither 6A-R nor 11C-R experienced a dramatic shift in the temporal progression of the IDC, nor did they develop artemisinin-resistance associated genotypes that have been previously observed *in vivo—*most notably, mutations in *pfk13*. In spite of these genotypic and phenotypic discrepancies, these derived parasite lines nonetheless provide a unique opportunity for future analyses of artemisinin resistance in the context of multiple genetic backgrounds[27,28]. The apparent lack of *pfk13* polymorphisms in 6A-R and 11C-R suggests that these parasites may serve as a model to study the relevant mechanisms driving the PfK13-independent artemisinin resistance phenotype newly emerging in Southeast Asia[48] and Africa[90].

The prerequisite of a genetic background and the possibility of “PfK13-independence” suggest that other genetic polymorphisms will contribute to the overall phenotype of artemisinin resistance that is currently in existence or will emerge in the future. Genomic profiling of 6A-R and 11C-R revealed unique sets of SNPs and CNVs that could represent such polymorphisms. Surprisingly, these polymorphisms did not involve genes with associations to any drug sensitivity phenotypes of malaria parasites reported in the past. The two exceptions include *pffp2a* and *pfprp22*. Pffp2a is a cysteine protease involved in hemoglobin digestion that is believed to mediate the activation of artemisinin presumably via the release of haemoglobin-derived iron[91]. Indeed Pffp2a can modulate artemisinin sensitivity in the ring stages[92], and a nonsense mutation in *pffp2a* has been previously found in an *in vitro*-derived artemisinin-resistant parasite line[36,37]. Hence the presence of the nonsense mutation the *pffp2a* likely contributes to artemisinin resistance in 6A-R. The amplification of *pfprp22* in both 6A-R and 11C-R on the common segment of chromosome 10 coincides with its duplication in another resistant parasite line derived from a D6 strain using artelinic acid[83,93]. However, this amplification at chromosome 10 had already been reported in naturally occurring infections including artemisinin sensitive parasites[75]. Hence its role in artemisinin resistance remains unclear. Here, we were able to substantiate the potential of the chromosome 14 CNV to influence the parasite’s sensitivity to artemisinin by the specific overexpression of two key genes in this region: *pftrx1* and *pfspp*. Both PfTrx1 and PfSpp play a role in the parasite’s antioxidant defense system and/or protein damage stress response. Thioredoxin 1 is a key enzyme in the *Plasmodium* NADPH-dependent thioredoxin system which is involved in the detoxification of reactive oxygen metabolites, redox regulation of transcription factors, and control of protein folding[77,78,94], while Signal Peptide Peptidase is a transmembrane protease component of the ER-associated degradation pathway, which is utilized by the parasite to cope with damaged or misfolded proteins[81]. Hence, the upregulation of *pftrx1* and *pfspp* likely supports the increased capacity of the UPR which, in eukaryotic cells, subsequently employs trafficking across cellular compartments, enzymatic processing of proteins to mediate their folding and degradation, and attenuation of translation to mitigate the ER workload[95,96,97]. Consistent with this model, 6A-R and 11C-R both demonstrated differential expression of genes related to translational control and the regulation of gene expression, including early translational events (initiation), binding and processing of messenger RNA, as well as transcriptional regulation via transcription factors and chromatin modification (see **Fig 3A, Tables A and B in S4 Table**). It has been previously shown that that *Plasmodium* is also able to cope with cellular stresses via translational repression involving the eif2α-mediated attenuation of global protein synthesis[97,98,99], and the association of mRNA with RNA-binding proteins that facilitate their stability (stress granules) or degradation (P-bodies)[98,99,100]. Transcriptional changes in many of these pathways were also observed in the *in vivo* isolates[34]. Taken together, this data represents a spectrum of SNPs and CNVs that may represent multiple, alternative genetic events that are yet to be observed or validated in the field but could emerge and spread in the (near) future. These could either deepen the existing *pfk13*-dependent artemisinin resistance phenotypes, or could give rise to new mechanisms compounding alternative genetic backgrounds of *P*. *falciparum* populations (e.g. Indian or African)[27,48,90].

## Materials and methods

### *In vitro* culture of *Plasmodium falciparum*

Two clonal parasite lines, named 6A and 11C, were previously derived from the *P*. *falciparum* 3D7 strain using limiting dilution[41] and subsequently used for *in vitro* drug selection. Continuous cultivation of parasites was performed as previously described[101]. Cultures were maintained in purified human red blood cells at 1–2% hematocrit, in RPMI 1640 medium (Gibco) supplemented with 0.25% Albumax I (Gibco), 2 g/L Sodium bicarbonate (Sigma), 0.1 mM hypoxanthine (Sigma), and 50 μg/L gentamicin (Gibco). Parasite cultures were kept at 37 ^o^ C with 5% CO_2_, 3% O_2_, and 92% N_2_ and treated twice with 5% (v/v) sorbitol (Sigma) every cycle to maintain stage synchronicity. Culture medium was replenished every 12–24 hours, and freshly washed uninfected red blood cells (RBC) was added to the culture as needed. Monitoring of parasitemia and parasite morphology was performed using microscopic evaluation of thin blood smears that were first fixed with methanol (Merck), and then stained with Giemsa (Sigma).

Ethical approval for the use of blood in this study was granted by the Institutional Review Board of the Nanyang Technological University. All of the blood utilized for the *in vitro* cultivation of parasites was derived from healthy adult volunteers, and extracted by trained personnel at the National University Hospital Blood Donation Center, Singapore. All donors provided their written informed consent.

### *In vitro* selection of artemisinin-resistant *Plasmodium falciparum*

6A and 11C parents were each divided into two parasite lines: one selection line (6A-R, 11C-R), which would be subjected to artemisinin selection, and one control line (6A and 11C), which would undergo mock treatment with dimethyl sulfoxide (DMSO) (Sigma). All parasite lines were synchronized at 4 HPI and diluted to a parasitemia (percentage of parasitized erythrocytes) of 2–5% prior to drug treatment. Each selection line was then pulse treated with a 900 nM artemisinin (Sigma) diluted in DMSO for four hours from 10–14 HPI; Control lines were also pulse treated in parallel with pure DMSO, for four hours at 10–14 HPI. During treatment, all parasites were kept at 2% hematocrit with 1 mL of parasitized blood. After treatment, the media containing artemisinin and DMSO were removed, and the parasite pellets washed twice with fresh media. Parasites were then resuspended in fresh media. Blood smears fixed with methanol and then stained with Giemsa were prepared for each parasite line 20–24 hours after washing to obtain post-treatment parasitemia as well as observe any morphological effects of drug treatment. During the initial phase of drug selection, artemisinin-treated parasites were allowed to recover to a viable parasitemia of at least 2% before artemisinin treatment. Once the parasite lines were able to consistently survive artemisinin pressure, they were maintained as synchronized cultures and subjected to pulse treatment with 900nM artemsinin from 10–14 HPI every other asexual cycle. Cultures were not kept away from artemisinin/DMSO treatment for more than three consecutive generations. Both sets of parasite lines were subjected to the same number of artemisinin and DMSO treatments throughout the course of drug selection, and at the same generations.

### *In vitro* drug sensitivity assay

#### Standard (72-hour) drug assay and 4-hour pulse drug assay

Ring-stage parasites growing in a tightly synchronous culture were synchronized by treatment with 5% (v/v) sorbitol at 2–4 HPI, and diluted down to 1% parasitemia, 2% hematocrit. They were then dispensed into 24-well plates containing 12–18 serially diluted concentrations of the drug being tested to make for a final parasitemia of 1%, at 1% hematocrit. For non-artemisinin antimalarial drugs quinine (QN) (Sigma), chloroquine (CQ) (Sigma), mefloquine (MEF) (Sigma) and pyrimethamine (PYR) (Sigma), parasites were incubated continuously for 72 hours with the drug starting from 6 HPI and into the next invasion cycle. For artesunate (ATS) (Sigma), dihydroartemisinin (DHA) (Sigma), dithiothreitol (DTT) (Merck), epoxomicin (EPX) (Sigma) and hydrogen peroxide (H_2_O_2_) (Merck), parasites were incubated in drug for 4 hours at 10–14 HPI, after which the cultures were washed twice with fresh media to remove the drug, and finally resuspended at 1% hematocrit and then allowed to reinvade. In the case of artemisinin, drug sensitivity was evaluated using the standard (72-hour) drug assay format, as well as the 4-hour pulse format at 10–14 HPI, 20–24 HPI and 30–34 HPI. Number of new, viable parasites in each well on the subsequent cycle after invasion was then evaluated by flow cytometry using a double staining method that utilizes Hoechst 33342 (Sigma) and dihydroethidium (Sigma) as previously described[102]. All assays involved technical duplicates per dose and was always done in parallel for 6A-R and 6A, and 11C-R and 11C at the same generations. Dose-response curves were plotted and fitted using the ‘drc’ package in R; IC50 and IC50_4hr_ values were obtained using the same package.

#### Ring-stage Survival assay (RSA)

The RSA was performed as previously described[45]. Briefly, tightly synchronous parasites are synchronized at 1–2 HPI and then incubated with 700 nM DHA for 6 hours, after which they were allowed to grow into the next invasion cycle for 66–72 hours. The amount of remaining viable parasites was measured with flow cytometry using Sybr Green I (Invitrogen) and MitoTracker Deep Red FM (Molecular Probes). All assays were carried out in biological triplicates, where each experiment involved technical duplicates per dose, and was always done in parallel for 6A-R and 6A, and 11C-R and 11C at the same generations.

#### Genome sequencing

Genomic DNA that was to be used for whole genome sequencing using the Illumina MiSeq platform was purified from parasitized RBCs using the Easy DNA kit (Invitrogen) according to the manufacturer’s specifications. Raw reads were mapped back to the *Plasmodium falciparum* 3D7 reference genome using the Burrows-Wheeler Alignment tool (BWA)[103], and reads having a mapping quality score < 30 were discarded. The sequence alignment data was then post-processed using SAMtools[104] and SNPs were called using SAMtools:mpileup. Only high quality intragenic SNPs where the non-reference allele has position coverage > 75% were included in the analysis. Hypervariable genes belonging to multi-gene families were not taken into consideration.

#### Microarray-based comparative genomic hybridization

Genomic DNA to be used for microarray-based comparative genomic hybridization (CGH) was isolated using the phenol-chloroform extraction method[105]. CGH was then carried out on DNA from 6A-R, 6A, 11C-R and 11C as previously described in detail[106,107]. Genomic DNA (3 μg) was first amplified and coupled with aminoallyl-dUTP (Biotium) using Klenow Fragment (3’- 5’exo-) enzyme (New England Biolabs) and random (N9) primers (1 mM). The reaction products were then purified using the MinElute PCR purification kit (Qiagen) following the manufacturer’s instructions. The Klenow reaction product (2–3 μg) was then labelled with either Cy5 (GE Healthcare) or Cy3 (GE Healthcare) in the presence of 0.1M NaHCO3 pH 9.0 for 4 hours in the dark, and then purified using the MinElute PCR purification kit (Qiagen). Equal amounts of Cy5-labelled sample DNA and Cy3-labelled reference DNA were then mixed with 2x Hybridization Buffer (Agilent Technologies) and deposited onto a microarray chip containing 10,367 probes covering over 5,000 genes in the *Plasmodium falciparum* genome. Microarrays were incubated at 65 ^o^ C in rotator oven (Agilent Technologies) for 20 hours and then scanned using the PowerScanner (Tecan). Background subtraction, normalization and filtering of raw signal intensities for each array was carried out using the R package ‘limma’[108], and detection of copy number alterations was carried out using the R package ‘GADA’ (Genomic Alteration Detection Analysis)[109].

### Transcriptional profiling

#### Sample collection for transcriptional profiling

In order to ensure tight synchrony, resistant and sensitive parasite cultures were subjected to five consecutive sorbitol treatments over three generations prior to harvesting samples for the first timepoint for each experiment.

#### IDC transcriptome

Starting from the early ring stage (4 HPI), six timepoints for each parasite line were harvested at 8-hour intervals over the course of the entire asexual blood stage.

#### Steady state ring-stage transcriptome

Parasitized RBCs were harvested during the mid-ring stage at the same timepoints that were targeted for arteminisinin selection. Six timepoints were collected at 2-hour intervals starting from 10 HPI until 20 HPI.

#### Ring-stage artemisinin treatment transcriptome

Transcriptional profiling of parasites under artemisinin pressure was also performed during the 4-hour window, and under the same drug concentration (900 nM) used for artemisinin selection. Highly synchronized parasites were exposed to artemisinin at 10 HPI, and RBC fractions were collected at 1 hour, 2 hours, and 4 hours after the start of treatment. In parallel, cultures treated with DMSO instead of artemisinin were grown and harvested under the exact same conditions to serve as controls.

#### RNA extraction

Total RNA was extracted from tightly synchronized parasite cultures using the TRIZol-Chloroform method as previously described in detail[107]. Briefly, the parasitized RBC pellet was resuspended in TRIzol reagent (Invitrogen). Chloroform (Fisher-Scientific) was then added, and the resulting aqueous phase was recovered and mixed with isopropanol to precipitate the RNA. The RNA was then resuspended in nuclease-free water and stored in aliquots at -80 ^o^ C.

#### cDNA synthesis, SMART amplification and microarray hybridization

The protocol for first strand cDNA synthesis, SMART amplification of double-stranded cDNA, and finally microarray hybridization was carried out using the protocol previously published by Bozdech *et al*.[107]. 500 ng of total RNA was used as the input for reverse transcription was carried out using SuperScript II (Invitrogen). The cDNA product—which represents the total cDNA pool—was then amplified using SMART-PCR using a mixture of aminoallyl-dUTP and dNTPs. 4 μg of SMART-amplified samples were then labelled with Cy5. On the other hand, the reference cDNA which was generated from pooling together RNA from all IDC stages of 3D7 was labelled with Cy3. Equal amounts of Cy5- and Cy3-labelled products were then deposited onto a microarray chip containing 10,265 probes covering over 5,000 genes in the *Plasmodium falciparum* genome. Background subtraction, normalization and filtering of raw signal intensities for each array were done using the R package ‘limma’[108]. The resulting log_2_ratio expression values of redundant probe sets representing a single gene were then averaged to obtain the mean expression for that particular gene. These gene expression datasets were then used for downstream analyses.

#### Data analysis for transcriptomic data

The IDC transcriptomes of each resistant and sensitive parasite lines were reconstituted using the Fast Fourier Transform method described previously[33,101]. Furthermore, the parasite age for each sample was subsequently estimated with respect to a reference IDC transcriptome (Dd2 strain) based on a Spearman Rank Correlation Coefficients as described by Mok *et al*.[33]. Significantly up- and downregulated genes were identified at p-value < 0.05 and FDR < 0.25. The p-values for each gene were calculated using pairwise student’s t-test and corrected by expression permutation (n = 1000) across timepoints. FDR was estimated by expression permutation across genes (n = 1000). Z-scores were also calculated for each gene according to the formula described in the previously published work by Mok *et al*. 2011[33]. In brief, it takes into account the ratio of the signal difference between resistant and sensitive parasite lines to noise, and rescaled to the number of time points. Genes were then ranked by z-scores and subjected to Gene Set Enrichment Analysis (GSEA) to identify differentially expressed functional pathways[110]. Enriched gene sets having a nominal p-value < 0.05 and FDR q value < 0.25 were considered to be statistically significant.

#### Plasmid construction

Three candidate genes (PF3D7_1454700, 6-phosphogluconate dehydrogenase (*pf6pgd*), PF3D7_1457200, Thioredoxin 1 (*pftrx1*), and PF3D7_1457000, Signal Peptide Peptidase (*pfspp*) were chosen for functional validation using a stable, episomal overexpression system. Full-length cDNA of each gene was first derived using gene-specific primers having the *NheI* restriction site on the 5’ end of the forward primer and the *BamHI* restriction site on the 5’ end of the reverse primer to allow for unidirectional cloning into the multiple cloning site (MCS). Each gene was inserted into pBcamR_3xHA vector[111] at the *BamHI/NheI* sites on the MCS upstream of the triple hemagglutinin (HA) sequence in order to obtain an HA-tagged protein product. The recombinant plasmids were then transfected into 3D7-6A parasites as previously described[112] and maintained via positive selection with 2.5 μg/mL blasticidin (BSD) (Sigma). A parasite line was also generated by transfecting 3D7-6A parasites with the empty pBcamR_3xHA plasmid and grown in BSD alongside the overexpression parasite lines to serve as vector control. Primers used for generating inserts are listed in **S8 Table**.

#### Quantitation of gene overexpression

To verify that the overexpression lines were indeed producing a surplus of each candidate gene, real-time quantitative PCR (relative quantification) was performed using gene-specific primers that are able to interrogate both the endogenous and episomal transcript, and using PF3D7_1218600 (Arginyl-tRNA synthetase) as the reference gene. The parasite line transfected with the empty pBcamR_3xHA vector was used as the control sample for ΔΔCt.

Total RNA was first extracted from synchronized parasite cultures using the TRIZol-Chloroform method as described previously[107]. First strand cDNA synthesis was first carried out on 50 ng of each transfectant line using oligo-dT-primed reverse transcription using SuperScript II Reverse Transcriptase (Invitrogen) according to the manufacturer’s instructions. The first-strand product was then used as the starting template for real-time qPCR using KAPA SYBR FAST qPCR Universal Master Mix. The ΔΔCt method was used to analyze the relative changes in expression level for each of the four candidate genes, where ΔΔCt = [(Ct of Sample Target Gene–Ct of Sample Reference Gene)–(Ct of Control Target Gene–Ct of Control Reference Gene)], and 2^-ΔΔCt^ is taken as the fold-change of the relative gene expression. All PCR reactions were performed in triplicates. Primers used for quantitative PCR are listed in **S9 Table**.

#### Western blot analysis

The parasitized RBC fraction was first lysed with 0.1% Saponin (Sigma) in 1 x PBS, and then washed three times with 1 x PBS. The resulting RBC-free parasite pellet was then subsequently resuspended in 2 x Laemmli Buffer mixed with 1x EDTA-free Protease Inhibitor (Roche), and then boiled at 100 ^o^ C for 10–15 mins. The supernatant containing the total soluble protein lysate was collected and identical amounts of protein lysate (100 μg) from each transfectant line was then loaded onto and separated using a 12% SDS-PAGE gel. To probe the protein of interest, an anti-HA mouse monoclonal antibody (Santa Cruz Biotechnology) at 1:2000 dilution was used, while an anti-β-actin mouse monoclonal antibody (Sigma) at 1:2000 was used as a loading control. An horseradish peroxidase (HRP)-conjugated goat anti-mouse antibody (Santa Cruz Biotechnology) at 1:2000 dilution was used to probe the anti-HA primary antibody, and an HRP-conjugated sheep anti-mouse antibody (GE Healthcare) at 1:2000 dilution was used against the anti-actin antibody. Detection was performed using the Immunocruz Western Blotting Luminol Reagent (Santa Cruz Biotechnology) according to the manufacturer’s specifications, and the chemiluminescent image was acquired using the Luminescent Image Analyzer LAS4000 System.

## Supporting information

S1 Fig Effects of a 4-hour pulse of 900 nM artemisinin on *Plasmodium* parasites at 10–14 HPI. Following a 4-hour pulse treatment with artemisinin of unselected 6A and 11C parasites from 10–14 HPI, Giemsa-stained smears were prepared at three timepoints: right after treatment, 14 hours after treatment and 24 hours after treatment. In parallel, smears of DMSO-treated parasites were also monitored at the same timepoints for comparison.
(TIF)

S2 Fig Stage-specific chemosensitivity profiling of *in vitro*-selected *P*. *falciparum* against artemisinin. Plots depict the mean dose-response curves between resistant and control parasite lines comparing their stage-specific sensitivity to a 4-hour artemisinin pulse at 20–24 HPI (IC50_20hpi/4hr_) and 30–34 HPI (IC50_30hpi/4hr_)**(A)**, and their artemisinin sensitivity across the IDC using a standard drug assay format (IC50)**(B)**. Drug assays were performed in biological triplicates.
(TIF)

S3 Fig Chemosensitivity profiling of *in vitro*-selected *P*. *falciparum* against other antimalarials and artemisinin-related compounds. Plots show mean dose-response curves comparing the ring-stage sensitivity (IC50_10hpi/4hr_) of 6A-R vs. 6A, and 11C-R vs. 11C to a 4-hour pulse of artemisinin derivatives dihydroartemisinin (DHA) and artesunate (ATS)**(A),** as well as artemisinin-related compounds hydrogen peroxide (H_2_O_2_), epoxomicin (EPX) and dithiothreitol (DTT)**(C)**. Mean dose-response curves comparing chemosensitivity of 6A-R vs. 6A, and 11C-R vs. 11C for quinine (QN), chloroquine (CQ), mefloquine (MEF) and pyrimethamine (PYR) using a standard drug assay format (IC50) were also obtained **(B).** MEF sensitivity was also evaluated in a previously in vitro selected T996-derived artemisinin-resistant parasite line. The histogram depicts the mean IC50_10hpi/4hr_ for T996-R and its nonselected parent, while the adjacent plot shows their corresponding mean dose-response curves **(D)**. Drug assays were performed in biological triplicates.
(TIF)

S4 Fig Transcriptional profiling of *in vitro*-selected *P*. *falciparum* across the IDC and under artemisinin pressure. Genome-wide transcriptional profiling was performed for all parasite lines across the asexual blood stage (IDC) and under artemisinin pressure. **(A)**Heatmaps represent the IDC transcriptomes of 6A-R, 6A, 11C-R and 11C across the IDC, over 6 timepoints (TP) and sampled at 8-hour intervals; Genes have been ordered according to the phase and frequency of expression using Fourier analysis. The right panel shows parasite age in HPI of each parasite line that was estimated by calculating the maximum Spearman rank correlation of the transcriptomic information at each timepoint with a reference IDC transcriptome. **(B)**Comparative transcriptomic analysis was also done on resistant vs sensitive parasites under a 4-hour pulse of artemisinin from 10–14 HPI. Heatmaps show significantly up- and downregulated genes (corrected p-value < 0.05, FDR < 0.25) in artemisinin-resistant parasites relative to their artemisinin-sensitive controls. Noted are the differentially expressed pathways between artemisinin-resistant and artemisinin-sensitive parasites (GSEA p < 0.05, FDR < 0.25). All genes from the total transcriptomic datasets were ranked by their z-score based on the difference in expression between resistant and sensitive lines, and used for GSEA. A more detailed list of differentially expressed genes and pathways can be found in **S2A File** and **Table A in S5 Table** (6A-R vs 6A), and **S2B File** and **Table B in S5 Table** (11C-R vs 11C). Data shown represents time-course transcriptomes over a single IDC.
(TIF)

S5 Fig Tracking CNV stability of *in vitro*-selected *P*. *falciparum*. To check for the stability of the CNVs identified, CNV profiles for 6A-R and 11C-R were assessed three times across multiple generations and continuous cultivation under artemisinin selection over the course of five months. Chromosome plots reflect the subtracted log_2_ratio of the artemisinin-resistant parasite lines relative to their control counterparts.
(TIF)

S6 Fig Ring-stage chemosensitivity profiling of *pftrx1-*, *pf6pgd-* and *pfspp-*overexpression transfectants against artemisinin. Plots depict mean dose-response curves against a 4-hour pulse of artemisinin at the ring stage for each of the *pftrx1*-, *pf6pgd*-, and *pfspp*- overexpression parasite lines, shown side by side with that of the vector control. Drug assays were performed in biological triplicates.
(TIF)

S1 Table IC50_10hpi/4hr_ values of 6A-R, 6A, 11C-R and 11C throughout the course of artemisinin selection. Numbers represent the IC50_10hpi/4hr_ values (mean ± standard deviation).
(PDF)

S2 Table Artemisinin IC50_20hpi/4hr_, IC50_30hpi/4hr_ and IC50 values of 6A-R, 6A, 11C-R. Numbers represent the IC50_4hr_ and IC50 values (mean ± standard deviation).
(PDF)

S3 Table IC values of 6A-R, 6A, 11C-R for other antimalarials (DHA, ATS, QN, CQ, MEF and PYR) and artemisinin-related compounds (H_2_O_2_, DTT and EPX). Numbers represent the IC50_4hr_ values (mean ± standard deviation) for DHA, ATS, H_2_O_2_, DTT and EPX, and the 72-hour IC50 values (mean ± standard deviation) against non-artemisinin derivatives (QN, CQ, MEF, PYR).
(PDF)

S4 Table Differentially expressed pathways between artemisinin-resistant and artemisinin-sensitive parasites at 10–20 HPI (mid to late ring stage). Differentially expressed pathways between 6A-R vs 6A **(Table A)** and 11C-R vs 11C **(Table B)** were identified using GSEA. Enriched gene sets with a p-value < 0.05 and FDR < 0.25 are considered statistically significant.
(PDF)

S5 Table Differentially expressed pathways between artemisinin-resistant and artemisinin-sensitive parasites under 900 nM artemisinin pressure from 10–14 HPI. Differentially expressed pathways between 6A-R vs 6A **(Table A)** and 11C-R vs 11C **(Table B)** were identified using GSEA. Enriched gene sets with a p-value < 0.05 and FDR < 0.25 are considered statistically significant.
(PDF)

S6 Table Copy number-amplified genes detected in 6A-R and 11C-R. Listed are genes that show an increase in copy number in resistant parasite lines relative to their sensitive controls. Significant differences in mRNA expression between resistant and sensitive parasite lines across the IDC were evaluated using pairwise student’s t-test and corrected by expression permutation (n = 1000) across timepoints. FDR was estimated by expression permutation across genes (n = 1000). Genes having a corrected p-value < 0.05 and FDR < 0.25 are considered to be significantly upregulated.
(PDF)

S7 Table IC50_10hpi/4hr_ values of overexpression and vector control parasite lines. Numbers represent the values (mean ± standard deviation).
(PDF)

S8 Table Primers used for PCR amplification of full-length cDNA inserts for generating overexpression constructs. (PDF)

S9 Table Primers used for quantitative real-time PCR amplification. (PDF)

S1 File Significantly up- and downregulated genes between artemisinin-resistant and artemisinin-sensitive parasites at 10–20 HPI (mid to late ring stage). Differentially expressed genes between 6A-R vs 6A **(S1A File)** and 11C-R vs 11C **(S1B File)** were identified using pairwise student’s t-test and corrected by expression permutation (n = 1000) across timepoints. FDR was estimated by expression permutation across genes (n = 1000). Significantly up- and downregulated genes were identified at corrected p-value < 0.05 and FDR < 0.25.
(XLSX)

S2 File Significantly up- and downregulated genes between artemisinin-resistant and artemisinin-sensitive parasites under 900 nM artemisinin pressure from 10–14 HPI. Differentially expressed genes between 6A-R vs 6A **(S2A File)** and 11C-R vs 11C **(S2B File)** were identified using pairwise student’s t-test and corrected by expression permutation (n = 1000) across timepoints. FDR was estimated by expression permutation across genes (n = 1000). Significantly up- and downregulated genes were identified at corrected p-value < 0.05 and FDR < 0.25.
(XLSX)
