# Supplementary figures and images for: Oxidative stress and protein damage responses mediate artemisinin resistance in malaria parasites
Source: PLoS Pathog. 2018 Mar 14;14(3):e1006930. doi: 10.1371/journal.ppat.1006930 (PMC5868857; doi:10.1371/journal.ppat.1006930)

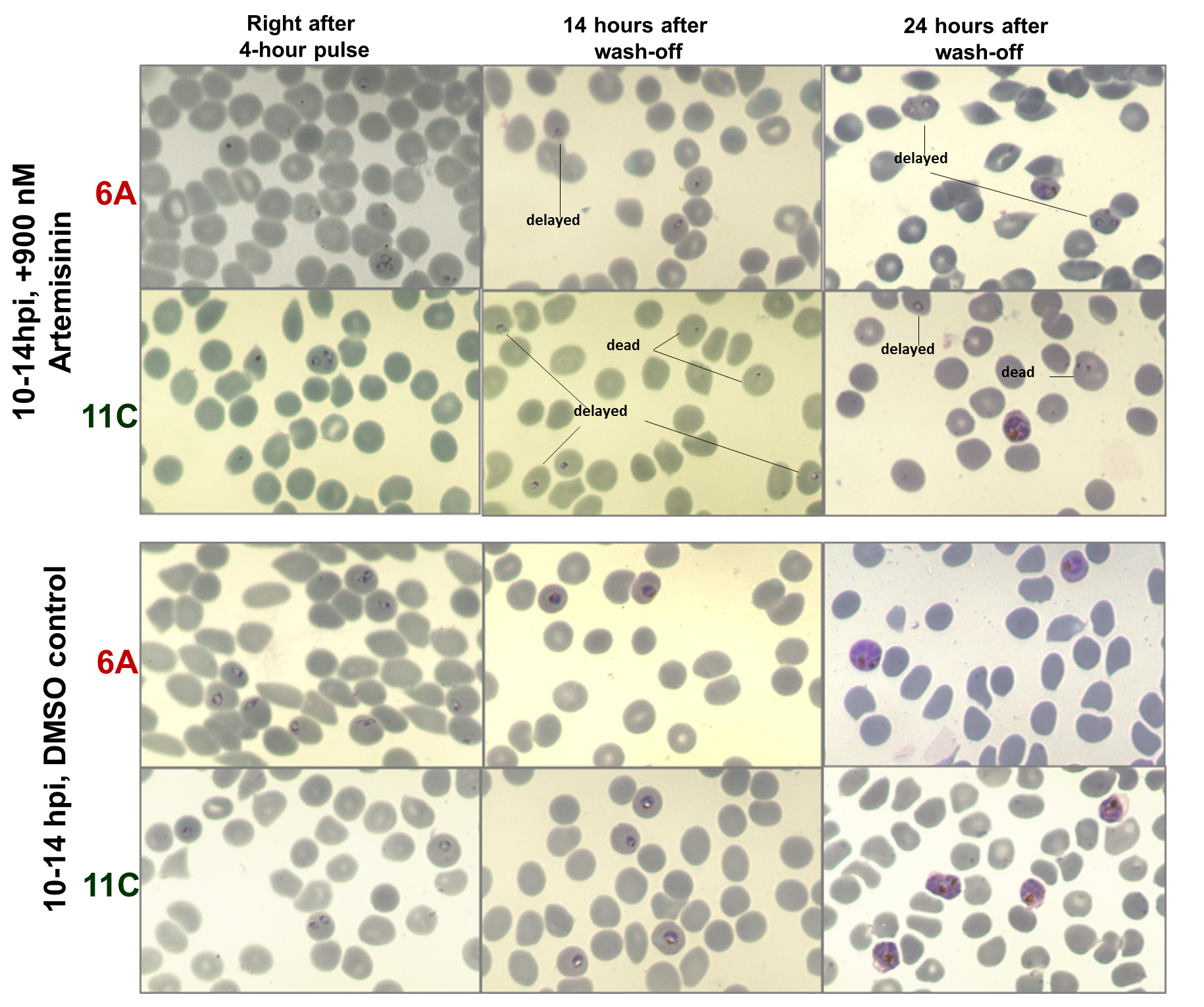

Supplement: S1 Fig — Following a 4-hour pulse treatment with artemisinin of unselected 6A and 11C parasites from 10–14 HPI, Giemsa-stained smears were prepared at three timepoints: right after treatment, 14 hours after treatment and 24 hours after treatment. In parallel, smears of DMSO-treated parasites were also monitored at the same timepoints for comparison. (TIF) [file ppat.1006930.s001.tif]

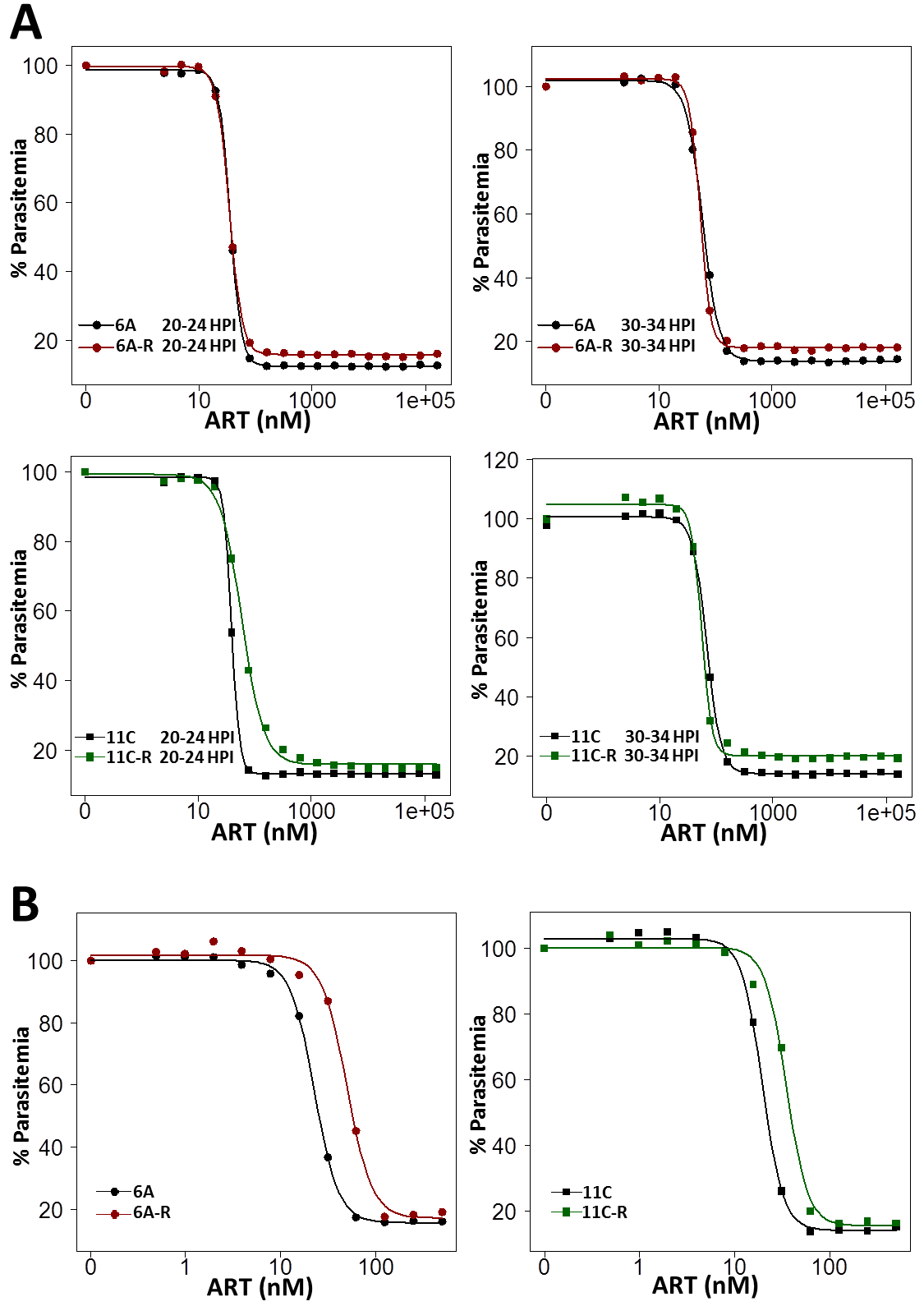

Supplement: S2 Fig — Plots depict the mean dose-response curves between resistant and control parasite lines comparing their stage-specific sensitivity to a 4-hour artemisinin pulse at 20–24 HPI (IC5020hpi/4hr) and 30–34 HPI (IC5030hpi/4hr)(A), and their artemisinin sensitivity across the IDC using a standard drug assay format (IC50)(B). Drug assays were performed in biological triplicates. (TIF) [file ppat.1006930.s002.tif]

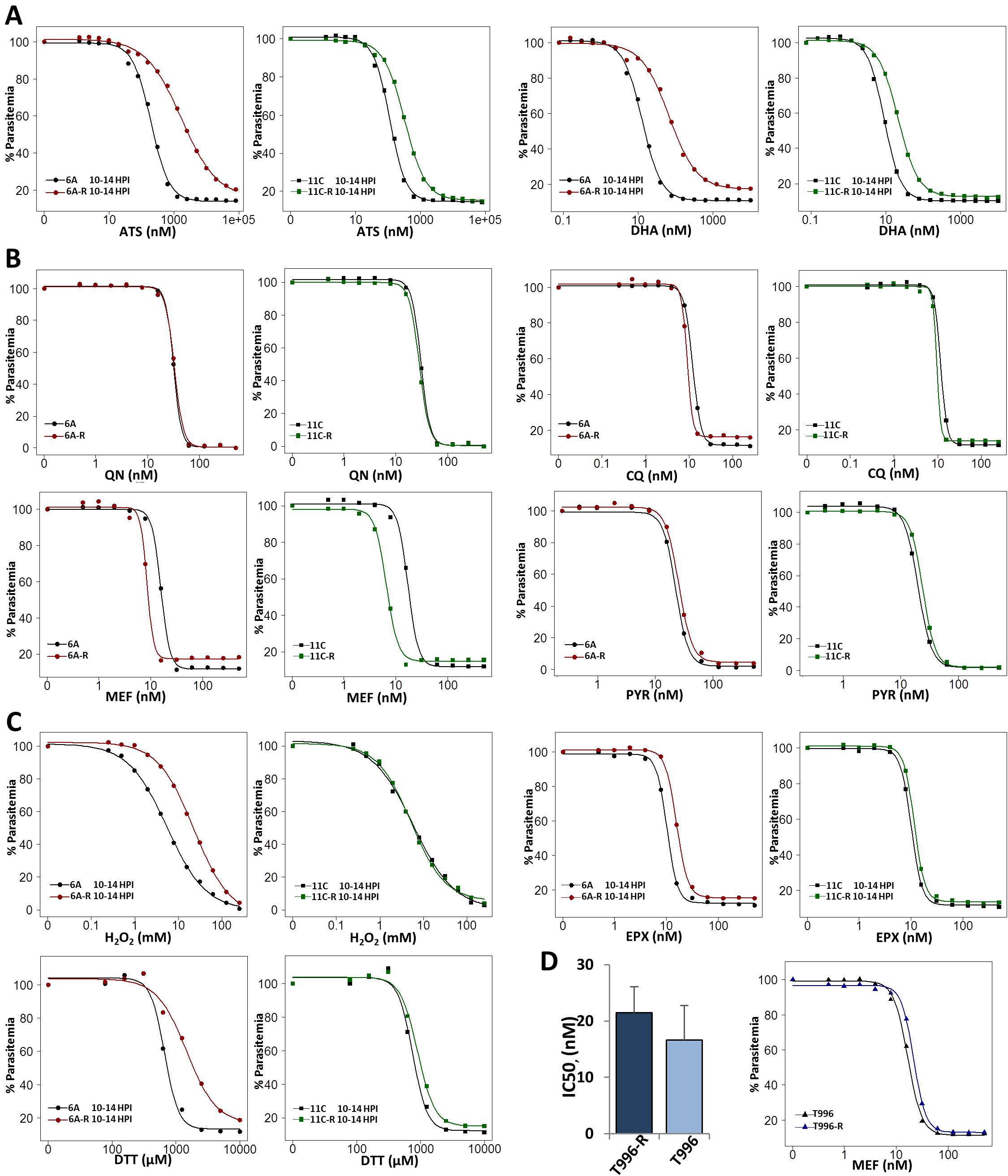

Supplement: S3 Fig — Plots show mean dose-response curves comparing the ring-stage sensitivity (IC5010hpi/4hr) of 6A-R vs. 6A, and 11C-R vs. 11C to a 4-hour pulse of artemisinin derivatives dihydroartemisinin (DHA) and artesunate (ATS)(A), as well as artemisinin-related compounds hydrogen peroxide (H2O2), epoxomicin (EPX) and dithiothreitol (DTT)(C). Mean dose-response curves comparing chemosensitivity of 6A-R vs. 6A, and 11C-R vs. 11C for quinine (QN), chloroquine (CQ), mefloquine (MEF) and pyrimethamine (PYR) using a standard drug assay format (IC50) were also obtained (B). MEF sensitivity was also evaluated in a previously in vitro selected T996-derived artemisinin-resistant parasite line. The histogram depicts the mean IC5010hpi/4hr for T996-R and its nonselected parent, while the adjacent plot shows their corresponding mean dose-response curves (D). Drug assays were performed in biological triplicates. (TIF) [file ppat.1006930.s003.tif]

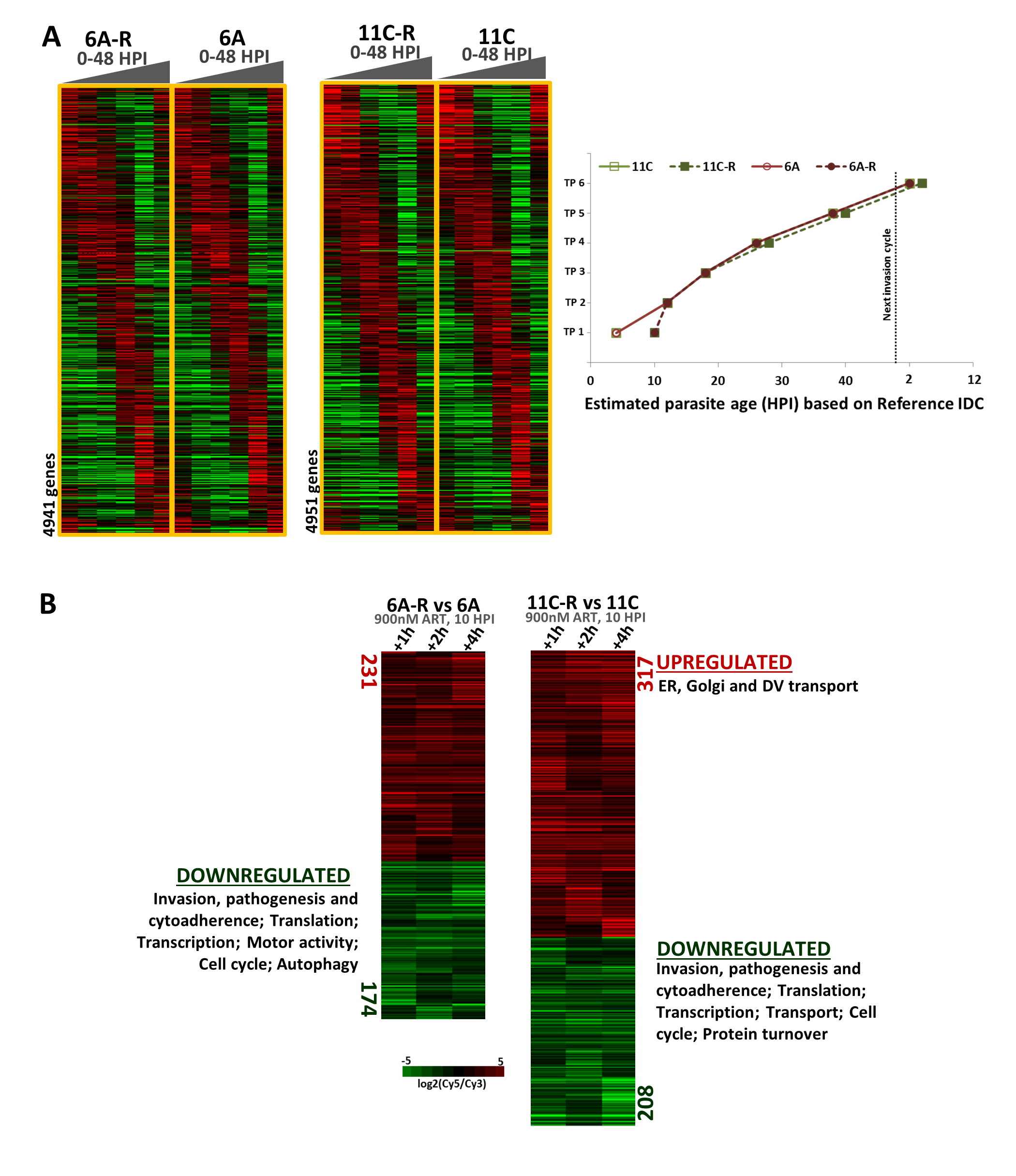

Supplement: S4 Fig — Genome-wide transcriptional profiling was performed for all parasite lines across the asexual blood stage (IDC) and under artemisinin pressure. (A)Heatmaps represent the IDC transcriptomes of 6A-R, 6A, 11C-R and 11C across the IDC, over 6 timepoints (TP) and sampled at 8-hour intervals; Genes have been ordered according to the phase and frequency of expression using Fourier analysis. The right panel shows parasite age in HPI of each parasite line that was estimated by calculating the maximum Spearman rank correlation of the transcriptomic information at each timepoint with a reference IDC transcriptome. (B)Comparative transcriptomic analysis was also done on resistant vs sensitive parasites under a 4-hour pulse of artemisinin from 10–14 HPI. Heatmaps show significantly up- and downregulated genes (corrected p-value < 0.05, FDR < 0.25) in artemisinin-resistant parasites relative to their artemisinin-sensitive controls. Noted are the differentially expressed pathways between artemisinin-resistant and artemisinin-sensitive parasites (GSEA p < 0.05, FDR < 0.25). All genes from the total transcriptomic datasets were ranked by their z-score based on the difference in expression between resistant and sensitive lines, and used for GSEA. A more detailed list of differentially expressed genes and pathways can be found in S2A File and Table A in S5 Table (6A-R vs 6A), and S2B File and Table B in S5 Table (11C-R vs 11C). Data shown represents time-course transcriptomes over a single IDC. (TIF) [file ppat.1006930.s004.tif]

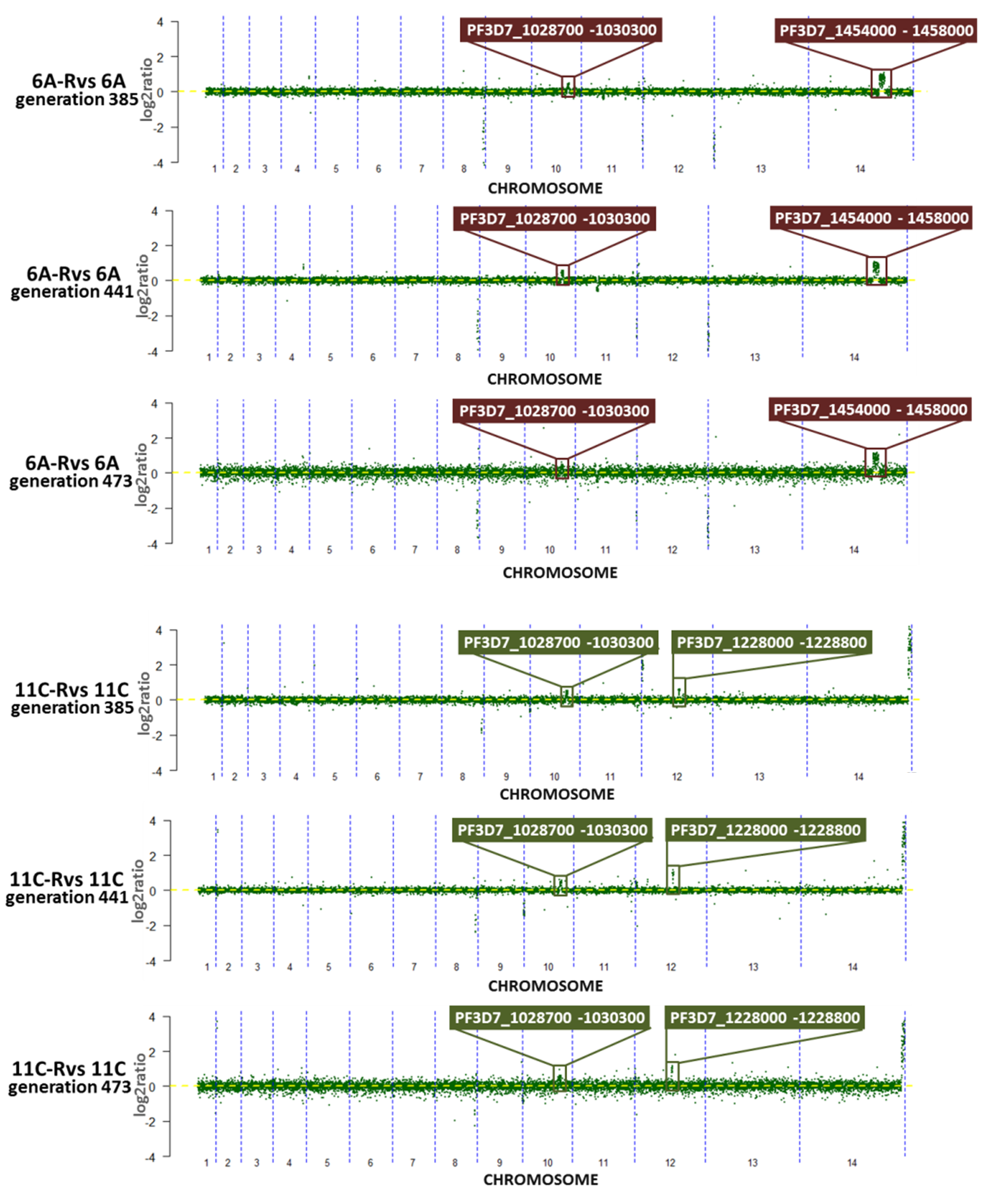

Supplement: S5 Fig — To check for the stability of the CNVs identified, CNV profiles for 6A-R and 11C-R were assessed three times across multiple generations and continuous cultivation under artemisinin selection over the course of five months. Chromosome plots reflect the subtracted log2ratio of the artemisinin-resistant parasite lines relative to their control counterparts. (TIF) [file ppat.1006930.s005.tif]

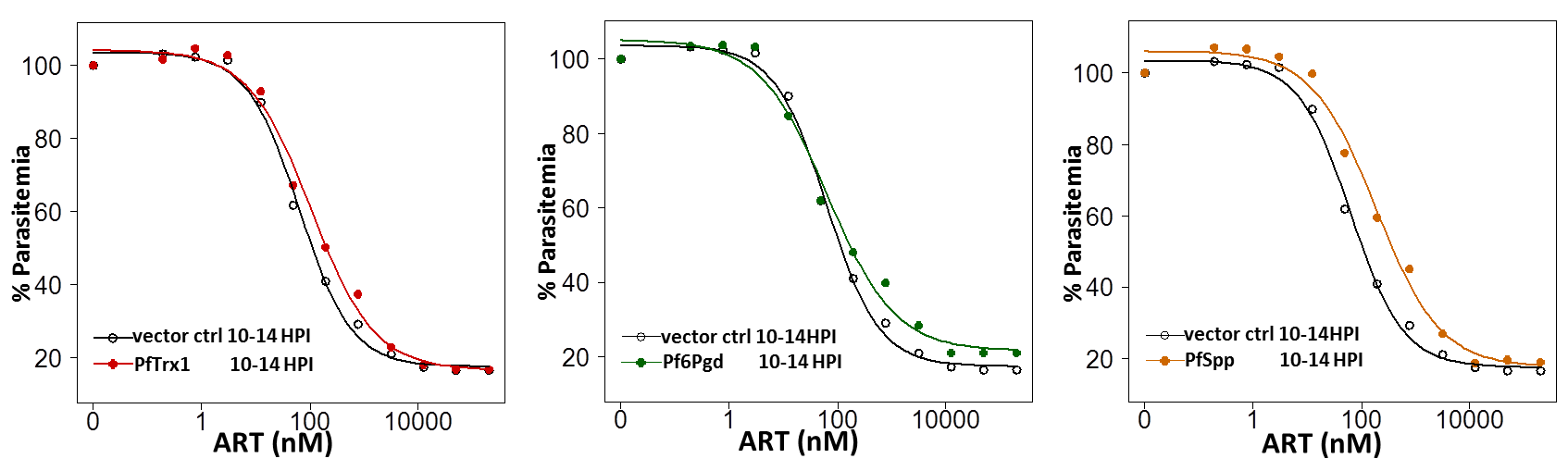

Supplement: S6 Fig — Plots depict mean dose-response curves against a 4-hour pulse of artemisinin at the ring stage for each of the pftrx1-, pf6pgd-, and pfspp- overexpression parasite lines, shown side by side with that of the vector control. Drug assays were performed in biological triplicates. (TIF) [file ppat.1006930.s006.tif]
