# Supplementary material for: Oxidative stress and protein damage responses mediate artemisinin resistance in malaria parasites
Source: PLoS Pathog. 2018 Mar 14;14(3):e1006930. doi: 10.1371/journal.ppat.1006930 (PMC5868857; doi:10.1371/journal.ppat.1006930)
Supplement: S1 Table — Numbers represent the IC5010hpi/4hr values (mean ± standard deviation). (PDF) [file ppat.1006930.s007.pdf]

SELECTION CYCLE	6A-R $IC50_{10hpi/4hr}$ (nM)	6A $IC50_{10hpi/4hr}$ (nM)	FOLD CHANGE IN $IC50_{10hpi/4hr}$
**6**	55.39 ± 17.15	18.13 ± 4.10	3
**8**	130.21 ± 14.86	41.71 ± 6.07	3
**37**	625.63 ± 140.28	234.79 ± 21.78	3
**38**	275.01 ± 14.62	86.09 ± 3.83	3
**40**	421.07 ± 23.04	64.36 ± 7.39	7
**100**	6,304.10 ± 591.35	92.76 ± 3.03	68
**112**	3,880.00 ± 193.11	137.37 ± 33.93	28
**175**	33,726.00 ± 8,540.44	84.66 ± 7.28	398
**237**	19,097.00 ± 5,330.88	97.44 ± 3.90	196

SELECTION CYCLE	11C-R $IC50_{10hpi/4hr}$ (nM)	11C $IC50_{10hpi/4hr}$ (nM)	FOLD CHANGE IN $IC50_{10hpi/4hr}$
**6**	35.73 ± 1.25	28.91 ± 0.42	1
**8**	521.91 ± 160.17	31.58 ± 3.62	17
**37**	3,052.00 ± 52.93	154.52 ± 8.01	20
**38**	2,059.60 ± 289.35	59.40 ± 2.76	35
**40**	2,458.64 ± 525.35	69.94 ± 3.94	35
**100**	3,134.80 ± 224.19	45.21 ± 13.46	69
**112**	2,144.50 ± 399.02	158.58 ± 20.43	14
**175**	1,587.30 ± 145.24	56.00 ± 6.26	28
**237**	1,982.50 ± 117.03	63.16 ± 3.17	31
