# Supplementary material for: Oxidative stress and protein damage responses mediate artemisinin resistance in malaria parasites
Source: PLoS Pathog. 2018 Mar 14;14(3):e1006930. doi: 10.1371/journal.ppat.1006930 (PMC5868857; doi:10.1371/journal.ppat.1006930)
Supplement: S2 Table — Numbers represent the IC504hr and IC50 values (mean ± standard deviation). (PDF) [file ppat.1006930.s008.pdf]

PARASITE	ART IC50$_{20hpi/4hr}$ (nM)	ART IC50$_{30hpi/4hr}$ (nM)	ART IC50 (nM)
**6A-R**	34.22 ±3.72	52.79 ± 7.15	49.75 ± 6.88
**6A**	35.30 ± 9.29	58.43 ± 21.9	22.62 ± 3.01
**11C-R**	58.62 ± 9.18	54.2 ± 5.92	35.07 ± 1.20
**11C**	38.47 ± 2.36	67.17 ± 8.01	19.60 ± 3.14
