# Supplementary material for: Oxidative stress and protein damage responses mediate artemisinin resistance in malaria parasites
Source: PLoS Pathog. 2018 Mar 14;14(3):e1006930. doi: 10.1371/journal.ppat.1006930 (PMC5868857; doi:10.1371/journal.ppat.1006930)
Supplement: S3 Table — Numbers represent the IC504hr values (mean ± standard deviation) for DHA, ATS, H2O2, DTT and EPX, and the 72-hour IC50 values (mean ± standard deviation) against non-artemisinin derivatives (QN, CQ, MEF, PYR). (PDF) [file ppat.1006930.s009.pdf]

PARASITE	DHA IC50$_{10hpi/4hr}$ (nM)	ATS IC50$_{10hpi/4hr}$ (nM)	QN IC50 (nM)	CQ IC50 (nM)	MEF IC50 (nM)	PYR IC50 (nM)	H_2O_2 IC50$_{10hpi/4hr}$ (mM)	DTT IC50$_{10hpi/4hr}$ (uM)	EPX IC50$_{10hpi/4hr}$ (nM)
**6A-R**	66.53 ± 30.89	1,693.10 ± 2043.36	30.29 ± 3.92	9.00 ± 0.21	8.39 ± 1.04	25.43 ± 0.60	22.64 ± 6.20	1,470.71 ± 134.39	15.59 ± 0.14
**6A**	12.81 ± 3.97	195.20 ± 20.49	31.56 ± 2.58	12.04 ± 2.77	15.59 ± 1.43	22.51 ± 2.31	5.43 ± 1.52	665.07 ± 81.27	10.19 ± 0.21
**11C-R**	21.37 ± 2.82	327.07 ±109.14	28.17 ± 2.24	9.55 ± 0.39	6.53 ± 0.37	23.95 ± 1.84	5.20 ± 0.88	897.86 ± 154.56	11.51 ± 0.64
**11C**	9.13 ± 3.43	119.64 ± 14.15	32.48 ± 31	11.76 ± 1.94	17.04 ± 2.71	19.88 ± 1.77	5.82 ± 1.83	754.15 ± 106.55	10.04 ± 0.51
