# Supplementary material for: Oxidative stress and protein damage responses mediate artemisinin resistance in malaria parasites
Source: PLoS Pathog. 2018 Mar 14;14(3):e1006930. doi: 10.1371/journal.ppat.1006930 (PMC5868857; doi:10.1371/journal.ppat.1006930)
Supplement: S4 Table — Differentially expressed pathways between 6A-R vs 6A (Table A) and 11C-R vs 11C (Table B) were identified using GSEA. Enriched gene sets with a p-value < 0.05 and FDR < 0.25 are considered statistically significant. (PDF) [file ppat.1006930.s010.pdf]

Table A

		UPREGULATED PATHWAYS	p-val	FDR q-val
Protein Turnover	MPM:	PROTEASOME-MEDIATED DEGRADATION OF NON-NATIVE ER PROTEINS	0.00	0.05
	MPM:	GENES CODING FOR COMPONENTS OF THE PROTEASOME DEGRADATION MACHINERY AND THEIR TIMED TRANSCRIPTION	0.02	0.12
	MPM:	PEPTIDASES AND PROTEASES	0.02	0.12
	MPM:	PROTEASOME-MEDIATED PROTEOLYSIS OF UBIQUINATED PROTEINS	0.03	0.21
	GO:	RESPONSE TO HEAT	0.02	0.16
	GO:	UBIQUITIN-DEPENDENT PROTEIN CATABOLIC PROCESS	0.03	0.23
	GO:	ER-ASSOCIATED PROTEIN CATABOLIC PROCESS	0.01	0.13
Oxidative Stress	MPM:	S-GLUTATHIONYLATED PROTEINS	0.00	0.04
	MPM:	PROTEINS TARGETED BY THE THIOREDOXIN SUPERFAMILY ENZYMES	0.03	0.17
	GO:	OXIDOREDUCTASE ACTIVITY	0.00	0.04
Transport	MPM:	GENES CODING FOR PROTEIN TRAFFIC RELATED PROTEINS	0.00	0.04
	MPM:	CLASSICAL COPII-MEDIATED VESICULAR TRANSPORT	0.01	0.13
	MPM:	SUBCELLULAR LOCATION OF ADAPTOR PROTEINS	0.01	0.13
	MPM:	GENES CODING FOR TRANSPORT PROTEINS	0.02	0.16
	MPM:	CLASSICAL CLATHRIN-MEDIATED VESICULAR TRANSPORT	0.04	0.21
	GO:	PROTEIN TRANSPORTER ACTIVITY	0.00	0.06
	GO:	INTRACELLULAR PROTEIN TRANSPORT	0.00	0.01
	GO:	VESICLE-MEDIATED TRANSPORT	0.01	0.13
Translational Elongation	MPM:	LOCALIZATION OF AMINOACYL-TRNA LIGASES IN THE THREE PROTEIN TRANSLATIONAL COMPARTMENTS	0.04	0.20
	GO:	TRANSLATIONAL ELONGATION	0.04	0.24
Mitochondrial Processes	MPM:	NUCLEAR GENES WITH MITOCHONDRIAL SIGNAL SEQUENCES	0.00	0.03
	MPM:	MITOCHONDRIAL ELECTRON FLOW	0.01	0.12
	MPM:	IMPORT OF PROTEINS INTO THE MITOCHONDRION	0.05	0.24
Cell cycle	GO:	REGULATION OF CELL CYCLE PROCESS	0.00	0.09

		DOWNREGULATED PATHWAYS	p-val	FDR q-val
Invasion, Pathogenesis and Cytoadherence	MPM:	FUNCTIONAL ANNOTATION OF MEROZOITE INVASION-RELATED PROTEINS	0.00	0.00
	MPM:	SUBCELLULAR LOCALIZATION OF PROTEINS INVOLVED IN INVASION	0.00	0.00
	MPM:	MEROZOITE LIGANDS THEIR ERYTHROCYTE RECEPTORS	0.01	0.08
	MPM:	PROTEIN-PROTEIN INTERACTIONS BETWEEN HUMAN ERYTHROCYTES AND PLASMODIUM FALCIPARUM	0.00	0.01
	MPM:	INTERACTIONS BETWEEN MODIFIED HOST CELL MEMBRANE AND ENDOTHELIAL CELL	0.00	0.00
	MPM:	*Pfemp1* DOMAIN ARCHITECTURES	0.00	0.00
	MPM:	ROSETTE FORMATION BETWEEN NORMAL AND INFECTED RBC	0.00	0.00
	GO:	CELL-CELL ADHESION	0.00	0.00
	GO:	CYTOADHERENCE TO MICROVASCULATURE MEDIATED BY SYMBIONT PROTEIN	0.00	0.00
	GO:	MODULATION BY SYMBIONT OF HOST ERYTHROCYTE AGGREGATION	0.00	0.00
	GO:	PATHOGENESIS	0.00	0.00
	GO:	ANTIGENIC VARIATION	0.00	0.00
Chromatin Modification	MPM:	PEPTIDES WITH CONFIRMED METHYLATED LYSINE RESIDUES	0.00	0.01
	MPM:	CHAPERONE-MEDIATED MODULATION OF NUCLEOSOME-HISTONE INTERACTIONS	0.03	0.12
Transcription	GO:	RNA METABOLIC PROCESS	0.03	0.14
	GO:	REGULATION OF TRANSCRIPTION DNA-DEPENDENT	0.05	0.16
Translation Initiation	MPM:	MATURATION AND EXPORT OF 60S AND 40S RIBOSOMAL SUBUNITS	0.03	0.10
	MPM:	RIBOSOMAL PARTICLES ALONG THE 60S ASSEMBLY PATHWAY	0.04	0.16
Motor activity	GO:	ACTIN BINDING	0.00	0.01
	GO:	MOTOR ACTIVITY	0.00	0.01
Phosphorylation	MPM:	PROTEIN PHOSPHORYLATION	0.01	0.04
	MPM:	THE PHOSPHOPROTEOME OF PLASMODIUM FALCIPARUM INFECTED RBCS	0.00	0.00
	MPM:	PROTEIN KINASE CODING GENES	0.00	0.00
Cell cycle	MPM:	PROTEINS PREDICTED TO BE INVOLVED IN CELL CYCLE REGULATORY NETWORK	0.03	0.10

Table B

		UPREGULATED PATHWAYS	p-val	FDR q-val
Protein Turnover	GO:	UBIQUITIN-DEPENDENT PROTEIN CATABOLIC PROCESS	0.03	0.15
	MPM:	GENERAL ROLES OF DEUBIQUITINASES	0.04	0.21
	MPM:	GENES CODING FOR COMPONENTS OF THE PROTEASOME DEGRADATION MACHINERY AND THEIR TIMED TRANSCRIPTION	0.05	0.20
Oxidative stress	MPM:	GLUTATHIONYLATED PROTEINS	0.00	0.06
Translation Initiation	MPM:	INITIATION OF TRANSLATION	0.00	0.00
	GO:	TRANSLATION	0.00	0.03
	GO:	TRANSLATIONAL INITIATION	0.01	0.10
	GO:	TRANSLATION INITIATION FACTOR ACTIVITY	0.01	0.04
	MPM:	RIBOSOMAL STRUCTURE	0.00	0.00
	MPM:	ORGANELLAR RIBOSOME ASSEMBLY PROTEINS AND THEIR PREDICTED TARGETING	0.00	0.03
	MPM:	GENES CODING FOR COMPONENTS INVOLVED IN RIBOSOME ASSEMBLY	0.01	0.06
	GO:	STRUCTURAL CONSTITUENT OF RIBOSOME	0.00	0.06
	MPM:	MATURATION AND EXPORT OF 60S AND 40S RIBOSOMAL SUBUNITS	0.05	0.20
Transcription	MPM:	TRANSCRIPTION ASSOCIATED PROTEINS IMPLICATED IN THE TRANSCRIPTIONAL MACHINERY	0.04	0.16
	MPM:	DNA BINDING PROTEINS WITH AP2 DOMAIN-S-	0.04	0.17
	MPM:	TRANSCRIPTION RELATED PROTEINS ENCODING GENES	0.03	0.14
	MPM:	MRNA-BINDING PROTEINS IN TROPHOZOITE AND SCHIZONTS	0.00	0.01
	MPM:	PREDICTED PROTEIN-PROTEIN INTERACTIONS OF PLAUSIBLE STRESS HELICASES	0.01	0.04
	MPM:	ENCODED PROTEINS THAT COULD BE CONSTITUENTS OF P-BODIES	0.05	0.22
Chromatin Modification	MPM:	PROTEINS CONTAINING HISTONE POST TRANSLATIONAL MODIFICATION-BINDING MODULES	0.00	0.01
	MPM:	HISTONE CHAPERONES	0.00	0.04
	MPM:	CHROMATIN MODIFYING PROTEINS	0.02	0.14
	MPM:	INTERACTOME OF THE SER-THR PROTEIN PHOSPHATASE TYPE 1	0.02	0.14
Phospholipid metabolism	MPM:	UTILIZATION OF PHOSPHOLIPIDS	0.02	0.14
	MPM:	FATTY ACIDS ELONGATION IN THE ENDOPLASMIC RETICULUM	0.03	0.19
Transport through the nucleus	MPM:	IMPORT AND EXPORT THROUGH THE NUCLEAR PORE	0.00	0.00
	MPM:	KARYOPHERIN-EXPORTIN-MEDIATED NUCLEAR EXPORT PATHWAYS	0.00	0.03
Exported proteins	MPM:	PARASITE ENCODED PROTEINS ASSOCIATED WITH THE MEMBRANE OF INFECTED ERYTHROCYTES	0.00	0.01
	MPM:	EXPORTED PROTEINS OF PLASMODIUM FALCIPARUM WITH CONFIRMED LOCATION	0.01	0.05
	MPM:	PROTEINS OF THE PARASITOPHOROUS VACUOLAR MEMBRANE	0.02	0.11
	MPM:	CHARACTERISTICS OF PLASMODIUM FALCIPARUM EXPORT PROTEINS THAT REMODEL INFECTED ERYTHROCYTE	0.02	0.11
Autophagy	MPM:	AUTOPHAGY AND AUTOPHAGY-RELATED PATHWAYS	0.00	0.03
	MPM:	THE ATG AUTOPHAGIC PATHWAY	0.00	0.04
Others	MPM:	PHOSPHOPROTEOME OF THE MEROZOITE	0.00	0.00
	MPM:	PROTEIN KINASE G-DEPENDENT PHOSPHORYLATION IN SCHIZONTS	0.04	0.15
	MPM:	DIFFERENTIAL PROTEOME ANALYSIS UNDER DRUG TREATMENT	0.01	0.13
	MPM:	S-NITROSYLATED PROTEINS	0.00	0.00

		DOWNREGULATED PATHWAYS	p-val	FDR q-val
Invasion, Pathogenesis, Cytoadherence	MPM:	INTERACTIONS BETWEEN MODIFIED HOST CELL MEMBRANE AND ENDOTHELIAL CELL	0.00	0.00
	MPM:	ROSETTE FORMATION BETWEEN NORMAL AND INFECTED RBC	0.00	0.00
	MPM:	*Pfemp1* DOMAIN ARCHITECTURES	0.00	0.01
	GO:	ANTIGENIC VARIATION	0.00	0.00
	GO:	CYTOADHERENCE TO MICROVASCULATURE MEDIATED BY SYMBIONT PROTEIN	0.00	0.00
	GO:	CELL-CELL ADHESION	0.01	0.09
	GO:	PATHOGENESIS	0.00	0.09
Transport	MPM:	GLYCOSYLPHOSPHATIDYLINOSITOL -GPI- ANCHOR BIOSYNTHESIS	0.01	0.09
	MPM:	SUBCELLULAR LOCATION OF ADAPTOR PROTEINS	0.00	0.12
Transcription	MPM:	RNA POLYMERASE II TRANSCRIBES MRNA	0.02	0.17
Translation elongation	GO:	TRANSLATION ELONGATION FACTOR ACTIVITY	0.03	0.19
Hemoglobin digestion	MPM:	HEMOGLOBIN DIGESTION	0.01	0.08
ATP synthesis	MPM:	ATP SYNTHASE COMPLEX	0.04	0.24
