# Supplementary material for: Oxidative stress and protein damage responses mediate artemisinin resistance in malaria parasites
Source: PLoS Pathog. 2018 Mar 14;14(3):e1006930. doi: 10.1371/journal.ppat.1006930 (PMC5868857; doi:10.1371/journal.ppat.1006930)
Supplement: S5 Table — Differentially expressed pathways between 6A-R vs 6A (Table A) and 11C-R vs 11C (Table B) were identified using GSEA. Enriched gene sets with a p-value < 0.05 and FDR < 0.25 are considered statistically significant. (PDF) [file ppat.1006930.s011.pdf]

Table A

DOWNREGULATED PATHWAYS			p-val	FDR q-val
Invasion, Pathogenesis and Cytoadherence	MPM:	PROTEIN-PROTEIN INTERACTIONS BETWEEN HUMAN ERYTHROCYTES AND PLASMODIUM FALCIPARUM	0.00	0.00
	MPM:	*Pfemp1* DOMAIN ARCHITECTURES	0.00	0.00
	MPM:	INTERACTIONS BETWEEN MODIFIED HOST CELL MEMBRANE AND ENDOTHELIAL CELL	0.00	0.00
	MPM:	ROSETTE FORMATION BETWEEN NORMAL AND INFECTED RBC	0.00	0.00
	GO:	PATHOGENESIS	0.00	0.00
	GO:	CELL-CELL ADHESION	0.00	0.00
	GO:	CYTOADHERENCE TO MICROVASCULATURE MEDIATED BY SYMBIONT PROTEIN	0.00	0.00
	GO:	ANTIGENIC VARIATION	0.00	0.00
Translation	MPM:	MATURATION AND EXPORT OF 60S AND 40S RIBOSOMAL SUBUNITS	0.04	0.25
Transcription	MPM:	TRANSCRIPTION ASSOCIATED PROTEINS IMPLICATED IN THE TRANSCRIPTIONAL MACHINERY	0.03	0.17
	MPM:	TRANSCRIPTION RELATED PROTEINS ENCODING GENES	0.03	0.18
	MPM:	PLASMODIUM FALCIPARUM CHROMATIN LANDSCAPE	0.01	0.18
Motor activity	GO:	ACTIN BINDING	0.00	0.00
	GO:	MOTOR ACTIVITY	0.00	0.01
	GO:	MICROTUBULE-BASED MOVEMENT	0.04	0.18
Cell cycle	MPM:	KINETOCHORES POWER CHROMOSOME MOVEMENTS IN MITOSIS	0.02	0.19
	MPM:	PROTEINS PREDICTED TO BE INVOLVED IN CELL CYCLE REGULATORY NETWORK	0.04	0.18
Autophagy	MPM:	AUTOPHAGY AND AUTOPHAGY-RELATED PATHWAYS	0.03	0.19

Table B

UPREGULATED PATHWAYS			p-val	FDR q-val
Transport	MPM:	TRANSPORTERS OF THE ER-GOLGI AND DIGESTIVE VACUOLE MEMBRANES	0.00	0.23

DOWNREGULATED GENES			p-val	FDR q-val
Invasion, Pathogenesis and Cytoadherence	MPM:	*Pfemp1* DOMAIN ARCHITECTURES	0.00	0.00
	MPM:	INTERACTIONS BETWEEN MODIFIED HOST CELL MEMBRANE AND ENDOTHELIAL CELL	0.00	0.00
	MPM:	ROSETTE FORMATION BETWEEN NORMAL AND INFECTED RBC	0.00	0.00
	MPM:	PROTEIN-PROTEIN INTERACTIONS BETWEEN HUMAN ERYTHROCYTES AND PLASMODIUM FALCIPARUM	0.00	0.01
	MPM:	FUNCTIONAL ANNOTATION OF MEROZOITE INVASION-RELATED PROTEINS	0.00	0.00
	MPM:	MEROZOITE LIGANDS THEIR ERYTHROCYTE RECEPTORS	0.00	0.00
	MPM:	SUBCELLULAR LOCALIZATION OF PROTEINS INVOLVED IN INVASION	0.00	0.00
	GO:	ANTIGENIC VARIATION	0.00	0.01
	GO:	PATHOGENESIS	0.00	0.00
	GO:	CELL-CELL ADHESION	0.00	0.00
	GO:	CYTOADHERENCE TO MICROVASCULATURE MEDIATED BY SYMBIONT PROTEIN	0.00	0.00
Translation	MPM:	RIBOSOMAL STRUCTURE	0.00	0.00
	MPM:	MATURATION AND EXPORT OF 60S AND 40S RIBOSOMAL SUBUNITS	0.00	0.00
	MPM:	GENES CODING FOR COMPONENTS INVOLVED IN RIBOSOME ASSEMBLY	0.00	0.00
	GO:	TRANSLATION INITIATION FACTOR ACTIVITY	0.01	0.05
	GO:	TRANSLATION	0.02	0.14
Transcription	MPM:	MODEL OF RNA POL II BIOGENESIS	0.04	0.12
	MPM:	SPLICING AND NON-SENSE-MEDIATED DECAY FACTORS	0.01	0.06
	MPM:	DNA BINDING PROTEINS WITH AP2 DOMAINS	0.04	0.14
Transport, Exported Proteins	MPM:	PROTEINS OF ERYTHROCYTE-DERIVED MICROVESICLES	0.00	0.01
	MPM:	COMPONENTS OF EXOSOMES	0.00	0.01
	MPM:	GENES CODING FOR GPI-ANCHORED MEMBRANE PROTEINS	0.00	0.03
	MPM:	PARASITE ENCODED PROTEINS ASSOCIATED WITH THE MEMBRANE OF INFECTED ERYTHROCYTES	0.02	0.07
	MPM:	ESTABLISHED AND PUTATIVE MAURER-S CLEFTS PROTEINS	0.02	0.10
	MPM:	PROTEINS OF THE PARASITOPHOROUS VACUOLAR MEMBRANE	0.03	0.13
	MPM:	EXPORTOME - COMPILED FROM VARIOUS SOURCES	0.00	0.00
Cell cycle	MPM:	PROTEINS PREDICTED TO BE INVOLVED IN CELL CYCLE REGULATORY NETWORK	0.01	0.03
Protein turnover	MPM:	LOCALIZATION AND FUNCTION OF SOME HSP40S AND HSP70S	0.00	0.00
Others	MPM:	PFWDR GENES	0.04	0.14
	MPM:	PROTEIN KINASE G-DEPENDENT PHOSPHORYLATION IN SCHIZONTS	0.04	0.13
	MPM:	S-NITROSYLATED PROTEINS	0.00	0.00
	MPM:	TOTAL PALMITOME OF PLASMODIUM FALCIPARUM	0.00	0.01
