# Supplementary material for: Oxidative stress and protein damage responses mediate artemisinin resistance in malaria parasites
Source: PLoS Pathog. 2018 Mar 14;14(3):e1006930. doi: 10.1371/journal.ppat.1006930 (PMC5868857; doi:10.1371/journal.ppat.1006930)
Supplement: S6 Table — Listed are genes that show an increase in copy number in resistant parasite lines relative to their sensitive controls. Significant differences in mRNA expression between resistant and sensitive parasite lines across the IDC were evaluated using pairwise student’s t-test and corrected by expression permutation (n = 1000) across timepoints. FDR was estimated by expression permutation across genes (n = 1000). Genes having a corrected p-value < 0.05 and FDR < 0.25 are considered to be significantly upregulated. (PDF) [file ppat.1006930.s012.pdf]

GENE ID	Description	6A-R vs 6A (0 – 48 HPI) p-val	6A-R vs 6A (0 – 48 HPI) FDR	11C-R vs 11C (0 – 48 HPI) p-val	11C-R vs 11C (0 – 48 HPI) FDR
PF3D7_1028700	merozoite TRAP-like protein	0.89	0.47	0.79	0.43
PF3D7_1028800	conserved Plasmodium protein, unknown function	0.83	0.43	0.99	0.50
PF3D7_1028900	conserved Plasmodium protein, unknown function	0.84	0.44	0.79	0.43
PF3D7_1029100	conserved Plasmodium protein, unknown function	0.68	0.38	0.48	0.30
PF3D7_1029200	conserved Plasmodium protein, unknown function	0.97	0.49	0.98	0.50
PF3D7_1029300	conserved Plasmodium protein, unknown function	0.01	0.02	0.04	0.05
PF3D7_1029400	conserved Plasmodium protein, unknown function	0.03	0.03	0.00	0.01
PF3D7_1029500	conserved Plasmodium protein, unknown function	0.18	0.14	0.01	0.01
PF3D7_1029600	adenosine deaminase	0.03	0.05	0.01	0.02
PF3D7_1029700	conserved Plasmodium protein, unknown function	0.01	0.01	0.29	0.20
PF3D7_1029800	RAP protein, putative	0.33	0.23	0.67	0.39
PF3D7_1029900	conserved Plasmodium protein, unknown function	0.54	0.34	0.63	0.39
PF3D7_1030000	transcription factor, putative	0.19	0.14	0.01	0.02
PF3D7_1030100	pre-mRNA-splicing factor ATP-dependent RNA helicase PRP22, putative	0.01	0.02	0.00	0.01
PF3D7_1030200	conserved Plasmodium protein, unknown function	0.48	0.30	0.38	0.26
PF3D7_1030300	conserved Plasmodium protein, unknown function	0.13	0.11	0.14	0.13
PF3D7_1228000	conserved Plasmodium protein, unknown function			0.31	0.21
PF3D7_1228100	leucine-rich repeat protein			0.26	0.19
PF3D7_1228200	conserved Plasmodium protein, unknown function			0.58	0.34
PF3D7_1228300	NIMA related kinase 1			0.93	0.47
PF3D7_1228400	conserved Plasmodium protein, unknown function			0.21	0.17
PF3D7_1228500	RNA pseudouridylate synthase, putative			0.22	0.15
PF3D7_1228600	merozoite surface protein 9			0.58	0.35
PF3D7_1228700	conserved Plasmodium protein, unknown function			0.19	0.14
PF3D7_1228800	conserved Plasmodium protein, unknown function			0.04	0.04
PF3D7_1454000	RNA-binding protein, putative	0.03	0.04		
PF3D7_1454100	tRNA intron endonuclease, putative	0.01	0.03		
PF3D7_1454200	conserved Plasmodium protein, unknown function	0.00	0.01		
PF3D7_1454300	serine/threonine protein kinase, putative	0.00	0.01		
PF3D7_1454400	aminopeptidase P	0.00	0.00		
PF3D7_1454500	nifU protein, putative	0.01	0.01		
PF3D7_1454600	mitochondrial ribosomal protein S11 precursor, putative	0.00	0.01		
PF3D7_1454700	6-phosphogluconate dehydrogenase, decarboxylating, putative	0.00	0.00		
PF3D7_1454800	conserved Plasmodium protein, unknown function	0.00	0.00		
PF3D7_1454900	conserved Plasmodium protein, unknown function	0.07	0.07		
PF3D7_1455000	protein phosphatase, putative	0.03	0.03		
PF3D7_1455100	protein phosphatase, putative	0.00	0.00		
PF3D7_1455200	methyltransferase, putative	0.22	0.16		
PF3D7_1455300	conserved Plasmodium protein, unknown function	0.04	0.04		
PF3D7_1455400	hemolysin, putative	0.13	0.11		
PF3D7_1455500	gamma-adaptin, putative	0.00	0.00		
PF3D7_1455600	ferlin, putative	0.41	0.25		
PF3D7_1455700	conserved Plasmodium protein, unknown function	0.03	0.04		
PF3D7_1455800	LCCL domain-containing protein	0.11	0.10		
PF3D7_1456000	transcription factor with AP2 domain(s)	0.03	0.03		
PF3D7_1456100	serine hydroxymethyltransferase, putative	0.50	0.29		
PF3D7_1456200	SNARE protein, putative	0.88	0.45		
PF3D7_1456300	conserved Plasmodium protein, unknown function	0.00	0.01		
PF3D7_1456400	conserved Plasmodium protein, unknown function	1.00	0.50		
PF3D7_1456500	conserved Plasmodium protein, unknown function	0.15	0.12		
PF3D7_1456600	mitochondrial ribosomal protein L28 precursor, putative	0.06	0.05		
PF3D7_1456700	conserved Plasmodium protein, unknown function	0.00	0.01		
PF3D7_1456800	V-type H(+)-translocating pyrophosphatase, putative	0.02	0.03		
PF3D7_1456900	conserved protein, unknown function	0.61	0.35		
PF3D7_1457000	signal peptide peptidase	0.00	0.01		
PF3D7_1457100	conserved Plasmodium protein, unknown function	0.00	0.00		
PF3D7_1457200	thioredoxin 1	0.01	0.02		
PF3D7_1457300	conserved Plasmodium protein, unknown function	0.00	0.01		
PF3D7_1457400	conserved Plasmodium protein, unknown function	0.00	0.00		
PF3D7_1457500	vacuolar protein sorting-associated protein 4	0.01	0.02		
PF3D7_1457600	conserved Plasmodium protein, unknown function	0.01	0.02		
PF3D7_1457700	large ribosomal subunit nuclear export factor, putative	0.01	0.02		
PF3D7_1457800	conserved Plasmodium protein, unknown function	0.01	0.03		
PF3D7_1457900	conserved Plasmodium protein, unknown function	0.00	0.01		
PF3D7_1458000	cysteine proteinase falcipain 1	0.02	0.03		
