# Supplementary material for: Oxidative stress and protein damage responses mediate artemisinin resistance in malaria parasites
Source: PLoS Pathog. 2018 Mar 14;14(3):e1006930. doi: 10.1371/journal.ppat.1006930 (PMC5868857; doi:10.1371/journal.ppat.1006930)
Supplement: S7 Table — Numbers represent the values (mean ± standard deviation). (PDF) [file ppat.1006930.s013.pdf]

Overexpression Parasite Line	ART IC50$_{10hpi/4hr}$ (nM)
**Vector ctrl**	63.48 ± 16.95
Pftrx1	105.68 ± 28.39
Pf6pgd	64.79 ± 39.54
Pfspp	186.08 ± 60.03
