# Supplementary material for: Oxidative stress and protein damage responses mediate artemisinin resistance in malaria parasites
Source: PLoS Pathog. 2018 Mar 14;14(3):e1006930. doi: 10.1371/journal.ppat.1006930 (PMC5868857; doi:10.1371/journal.ppat.1006930)
Supplement: S8 Table — (PDF) [file ppat.1006930.s014.pdf]

GENE	NAME	SEQUENCE (5'-3')
**PF3D7_1454700**, *Pf6pgd*	6pgd_BamHI	ATGCGGATCCATGTGTGATATTGGTTTGATAGGT
	6pgd_NheI	ATGCGCTAGCCCACAAGGTGTGATAATTTCC
**PF3D7_1457200**, *Pftrx1*	Trx_BamHI	ATCGGGATCCATGGTAAAAATTGTAACTAGTCAA
	Trx_NheI	ATCGGCTAGCAGCTGCGTATTTTTCGATAAG
**PF3D7_1457000**, *Pfspp*	Spp_BamHI	ATCGGGATCCATGAATTTATTAAAATTAATTGGA
	Spp_NheI	ATCGGCTAGCTTTATTGGTAATTCTTTTTTTTGC
