# Supplementary material for: Oxidative stress and protein damage responses mediate artemisinin resistance in malaria parasites
Source: PLoS Pathog. 2018 Mar 14;14(3):e1006930. doi: 10.1371/journal.ppat.1006930 (PMC5868857; doi:10.1371/journal.ppat.1006930)
Supplement: S9 Table — (PDF) [file ppat.1006930.s015.pdf]

GENE	NAME	SEQUENCE (5'-3')
**PF3D7_1454700**, *Pf6pgd*	6pgd_F	TGATGGTGGGAATGAATGG
	6pgd_R	ATTCCCTGAGGAACCTGGAC
**PF3D7_1457200**, *Pftrx1*	Trx_F	TTTTGCTGAATGGTGTGGAC
	Trx_R	TTAATGCTGAGTCGTTTGCTC
**PF3D7_1457000**, *Pfspp*	Spp_F	TTCCCAGTTTCGAGTGATCC
	Spp_R	GAAGAGCTGGTTGAGGATGTTC
**PF3D7_1218600**	R-tRNA_synth_F	GAGTACCCCAATCACCTACA
	R-tRNA_synth_R	AAGAGATGCATGTTGCTCATTT
